# Triacylglycerol Storage in Lipid Droplets in Procyclic *Trypanosoma brucei*

**DOI:** 10.1371/journal.pone.0114628

**Published:** 2014-12-10

**Authors:** Stefan Allmann, Muriel Mazet, Nicole Ziebart, Guillaume Bouyssou, Laetitia Fouillen, Jean-William Dupuy, Marc Bonneu, Patrick Moreau, Frédéric Bringaud, Michael Boshart

**Affiliations:** 1 Fakultät für Biologie, Genetik, Ludwig-Maximilians-Universität München, Biozentrum, Martinsried, Germany; 2 Centre de Résonance Magnétique des Systèmes Biologiques (RMSB), Unité Mixte de Recherche 5536, Université de Bordeaux/Centre National de la Recherche Scientifique (CNRS), Bordeaux, France; 3 Laboratoire de Biogenèse Membranaire, Unité Mixte de Recherche 5200, Université de Bordeaux/Centre National de la Recherche Scientifique (CNRS), Institut National de la Recherche Agronomique (INRA) Bordeaux, Villenave d’Ornon, France; 4 Centre de Génomique Fonctionnelle, Plateforme Protéome, Université de Bordeaux, Bordeaux, France; University of Hull, United Kingdom

## Abstract

Carbon storage is likely to enable adaptation of trypanosomes to nutritional challenges or bottlenecks during their stage development and migration in the tsetse. Lipid droplets are candidates for this function. This report shows that feeding of *T. brucei* with oleate results in a 4–5 fold increase in the number of lipid droplets, as quantified by confocal fluorescence microscopy and by flow cytometry of BODIPY 493/503-stained cells. The triacylglycerol (TAG) content also increased 4–5 fold, and labeled oleate is incorporated into TAG. Fatty acid carbon can thus be stored as TAG in lipid droplets under physiological growth conditions in procyclic *T. brucei*. β-oxidation has been suggested as a possible catabolic pathway for lipids in *T. brucei*. A single candidate gene, *TFEα1* with coding capacity for a subunit of the trifunctional enzyme complex was identified. *TFEα1* is expressed in procyclic *T. brucei* and present in glycosomal proteomes, Unexpectedly, a *TFEα1* gene knock-out mutant still expressed wild-type levels of previously reported NADP-dependent 3-hydroxyacyl-CoA dehydrogenase activity, and therefore, another gene encodes this enzymatic activity. Homozygous Δ*tfeα1*/Δ*tfeα1* null mutant cells show a normal growth rate and an unchanged glycosomal proteome in procyclic *T. brucei*. The decay kinetics of accumulated lipid droplets upon oleate withdrawal can be fully accounted for by the dilution effect of cell division in wild-type and Δ*tfeα1/*Δ*tfeα1* cells. The absence of net catabolism of stored TAG in procyclic *T. brucei*, even under strictly glucose-free conditions, does not formally exclude a flux through TAG, in which biosynthesis equals catabolism. Also, the possibility remains that TAG catabolism is completely repressed by other carbon sources in culture media or developmentally activated in post-procyclic stages in the tsetse.

## Introduction

Lipid droplets (LD) are dynamic organelles and conserved throughout prokaryotic and eukaryotic organisms [1]. The dynamic nature and interactions with other subcellular compartments are poorly understood [2]. They are heterogeneous particles bounded by a phospholipid monolayer also containing glycolipids and sterols. The core inside this monolayer contains triacylglycerols (TAG), diacylglycerols (DAG) and sterol esters. The composition varies between organisms and also cell types. The size of the particles ranges between 50 nm and 200 µm, the latter found in adipocytes. The monolayer contains specific proteins that are involved in biogenesis of the LD and mobilization of the stored lipids. LDs form or accumulate in response to starvation and various other stresses. In addition to carbon storage, a role in intracellular lipid trafficking or membrane biogenesis [3]–[6] was found in yeast as well as in mammalian cells. In *D. melanogaster* embryos intracellular repositioning has been reported during development [7]. In trypanosomes, the biogenesis of LDs seems to be regulated by a specific protein kinase [8], yet their function in metabolism of the organisms is unknown.

Carbon storage requires a pathway to catabolize the stored TAG. β-oxidation converts fatty acids (FA) into acetyl-CoA building blocks. This starts with the release of FA from TAG by a lipase followed by its activation in the cytosol by a long-chain fatty acyl-CoA synthetase (EC 6.2.1.3), giving rise to a fatty acyl-CoA ester. This ester then diffuses (<10 carbons) or is transported into the mitochondrion. Four subsequent steps produce acyl-CoA(n-2) and acetyl-CoA. The acetyl-CoA is oxidized to carbon dioxide, resulting in ATP production in the electron transport chain. In mammalian cells long chain fatty acids (n>22 carbons) are processed first within the peroxisomes, and the shortened acyl-CoA molecules moved to the mitochondrion. The *T. brucei* peroxisome-like organelles harbour glycolysis and thus are called glycosomes. Two enzymatic activities, enoyl-CoA hydratase (EC4.2.1.17) and 3-hydroxyacyl-CoA dehydrogenase (1.1.1.35), that are part of the trifunctional enzyme complex (TFE) of β-oxidation, have been identified and apparently localized to this organelle [9]. This suggested the parasites capability of FA degradation.

Storage and later utilization of FAs in starvation periods helps cells or organisms to survive changing environments and nutritional bottlenecks. This applies to parasitic organisms like *Trypanosoma brucei* during their life cycle in different host and vector environments. The causative agent of African Trypanosomiasis has a digenetic life cycle in a mammalian host and tsetse flies of the *Glossina spp.* as vector. While residing in the mammalian bloodstream the nutritional environment is homeostatic. In contrast, during the complex development in the insect vector [10] that involves migration through different organs, the parasite is challenged by changing carbon sources, oxidative stress [11] or different pH values [12]. This is particularly important during migrating from the midgut towards the salivary gland. Crossing the parasite-crowded proventriculus area of the foregut to reach the esophagus requires high parasite motility [13], depending on energy. Therefore, *T. brucei* may need energy stores for development within the insect host. This hypothesis is supported by electron microscopical detection of large LDs within the stumpy bloodstream and procyclic forms, while LD size was considerably reduced in parasites isolated from the proventriculus, and few or no LDs were detected in parasites isolated from the salivary glands [14]. This suggests a physiological role of LDs during developmental progression. LDs may form in the proliferating midgut stages and lipid stores might be utilized during the migration through the proventriculus towards the salivary glands. In agreement with this view, it has been shown that the procyclic forms take up fatty acids at a much faster rate than BSF [15].

Here we show uptake of fatty acids and their storage in LDs under physiological conditions, and followed the decay of LDs. A putative step of β-oxidation was investigated by reverse genetic tools.

## Experimental Procedures

### Trypanosome Culture and Transfections

The procyclic form of *T. brucei* AnTat 1.1 and EATRO1125 was cultured at 27°C in SDM79 medium containing 10% (v/v) heat-inactivated fetal calf serum and 35 µg/ml hemin [16]. The SDM79 used for glucose-depleted conditions was either prepared with normal FCS resulting in about 0.5 mM residual glucose (SDM79-Glu) or additionally preconditioned to fully consume glucose (SDM79GluFree). The SDM79GluFree medium was prepared by growing WT procyclic trypanosomes (5×10^6^ cells/ml) in glucose-free SDM79 supplemented with 20% FCS, during 3 days to late log phase (2×10^7^ cells/ml), then the spent medium was filtered and completed with one volume of fresh glucose- and FCS-free SDM79. In both media the addition of 50 mM N-acetylglucosamine (GlcNAc) was added to inhibit residual glucose import [17]–[19]. Oleate feeding was performed with 400 µM oleate complexed with BSA. The SDM79 medium containing oleate was prepared as described in [20]. The EATRO1125 procyclic form cell line constitutively expressing the T7 RNA polymerase gene and the tetracycline repressor under the control of a T7 RNA polymerase promoter for tetracycline-inducible expression (EATRO1125.T7T) [21], was the recipient of all transfections. Transfection and selection in SDM79 medium containing combinations of hygromycin B (25 µg/ml), neomycin (10 µg/ml), blasticidin (10 µg/ml), phleomycin (5 µg/ml) and puromycin (1 µg/ml) is described in [22].

### Fluorescence Microscopy

This protocol was carried out as described previously [23] with minor modifications. 1×10^7^ procyclic forms were fixed in 2% formaldehyde at 4°C, then washed three times with PBS for 5 min at 4°C. The fixed cells were attached to silanized coverslips by sedimentation and permeabilized with 0.2% NP-40 in PBS for 10 min at room temperature for BODIPY 493/503 (Molecular Probes). For nile red staining permeabilization was not necessary. Staining of lipid droplets was done with 1 µg/ml nile red or 5 µg/ml BODIPY for 30 min at RT. Cells were mounted in antifade solution (Vectashield) and analyzed by confocal laser scanning microscopy (CLSM) with a Leica SP5 CLSM microscope. Microscope settings were: 405 nm diode laser at 20%, Argon laser at 20% power and sequential scanning settings for PMT1: 420–473 nm, for PMT3: 498–564 nm. Stacks have been acquired with 0.5 µm step size and a total thickness of 5–8 µm.

### Flow Cytometry

We adapted BODIPY 493/503 staining for *T. brucei* which is widely used in the mammalian field [20] and has also been used for another kinetoplastid [24]. This dye gives the advantage of a higher specificity for nonpolar lipids and is compatible with multicolor imaging. It enables the analysis by flow cytometry, as there is only one emission spectrum and not two overlapping spectra as for nile red, where the binding to polar or nonpolar lipids creates a chromatic shift [20]. 1×10^7^−2×10^7^ procyclic cells were harvested and washed once in cold PBS (10 min, 900 g, 4°C). The cells were resuspended in 500 µl PBS and were fixed by addition of 500 µl 4% paraformaldehyde in PBS at 4°C for 2 h or over night. After PFA treatment all following centrifugations were carried out at 500 g, 4°C for 10 min. Cells were washed twice with PBS/2 mM EDTA. Permeabilization was done with 0.2% NP-40 in PBS for 15 min at RT. Cells were then washed once with 1 ml PBS/2 mM EDTA. Pellets have been resuspended in 400 µl PBS/2 mM EDTA containing 5 µg/ml BODIPY493/503 and incubated for 30 min at RT in the dark. Cells were pelleted and resuspended in 1 ml PBS/2 mM EDTA and analyzed with a BD FACS Calibur flow cytometer (488 nm Laser).

### Labeling with [1-^14^C]-oleate and lipid analysis

[1-^14^C]-oleate feeding was performed as follows: 10^8^ cells in the late exponential phase were incubated for 30 min, 1 h, 2 h and 8 h in 5 ml of SDM79 medium as indicated above and containing 6 µM [1-^14^C]-oleate (58.2 mCi/mmol, Perkin-Elmer SAS, Courtaboeuf, France) and 400 µM unlabeled oleate complexed with BSA. The SDM79 medium containing oleate was prepared as described in [20]. Subsequently, lipids were extracted by chloroform:methanol (2∶1, v/v) for 30 min at room temperature, and then washed three times with 0.9% NaCl. The solvent was evaporated and lipids were dissolved in an appropriate volume of chloroform/methanol (1∶1, v/v). To determine the labeling of total phospholipids and neutral lipids, the lipid extracts were loaded onto HPTLC plates (60F254, Merck) with a CAMAG Linomat IV and developed in hexane/ethylether/acetic acid (90∶15∶2, v/v). Total phospholipids (start), diacylglycerols (DAG, R_F_ 0.08), free fatty acids (FFA, R_F_ 0.29), triacylglycerols (TAG, R_F_ 0.50) and esters (R_F_ 0.90) were separated. Lipids were identified by co-migration with known standards and lipid radioactivity was determined with a Storm 860 (GE Healthcare) phosphorimager.

### TAG quantification by HPTLC

Lipid extracts were prepared as indicated above. To determine the amount of TAG, the lipid extracts were loaded onto HPTLC plates developed in hexane/ethylether/acetic acid (90∶15∶2, v/v) as indicated above. TAG amounts were quantified by densitometry using a CAMAG TLC scanner 3 as described in [25].

### TAG species identification by ESI/MS/MS

Prior to the MS analysis, the lipid extracts were resuspended with a mixture of chloroform/methanol 1/1 (v/v) containing 0.2% formic acid+0.028% NH_3_. Shotgun analysis was performed on a QTrap 5500 (ABSciex). Analyses were performed with neutral loss scans in positive mode. Nitrogen was used as curtain gas (set to 15), gas1 (set to 20) and gas2 (set to 0). Needle voltage was at +5,500 V without needle heating; the declustering potential was set at +40 V. The collision gas was also nitrogen and collision energy was adjusted to +40 eV. Samples were analyzed in duplicate. Triacylglycerols were identified and quantified using the Lipidview (ABSciex). Lipid species were quantified by normalizing the intensities of their peaks to the intensity of the peaks of the internal standard (TAG17∶0/17∶0/17∶0) spiked into the sample.

### NADPH-dependent 3-hydroxyacyl-CoA dehydrogenase activity in cell lysates and glycosome enriched fractions

For total lysates, cells were washed in PBS and lysed by sonication (5 sec at 4°C) in phosphate buffer (100 mM, pH 6.2). A subcellular fraction enriched in glycosomes was prepared by differential centrifugation of WT procyclic cells and of Δ*tfeα1/*Δ*tfeα1* cells as described in [28], after homogenizing the cells with silicon carbide as grinding material. Briefly, 5×10^9^ cells were washed once in 50 m of STE (25 mM Tris, 1 mM EDTA, 250 mM sucrose pH 7.8). After centrifugation, the pellet was resuspended in 0.5 m of homogenization buffer (STE supplemented with ‘Complete EDTA-Free’ protease-inhibitor cocktail, Roche Applied Science, Mannheim, Germany) and ground in a pre-chilled mortar with 1.5 gr of wet-weight silicon carbide per gr of cell pellet. The cells were microscopically checked for at least 90% disruption. The cell lysate was diluted in 7 ml of homogenization buffer, centrifuged at 1000 g and then at 5000 g for 10 min each, at 4°C. The supernatant was centrifuged at 33,000 g for 10 min at 4°C to yield the glycosome-enriched pellet, which was resuspended in 200 µl of phosphate buffer (100 mM, pH 6.2) and lysed by sonication (5 sec at 4°C) prior to activity assays. The GPDH activity and the 3-hydroxyacyl-CoA dehydrogenase activity were measured as described before in references [26] and [27] respectively. For the NADH-dependent and NADPH-dependent 3-hydroxyacyl-CoA dehydrogenase activities C4 substrates were used.

### Phylogenetic reconstruction

Sequences belonging to the TFEα2 group, including the mitochondrial Human sequence (P40939), were collected from a recent analysis [29]. Representative prokaryotic and eukaryotic sequences belonging to the TFEα1 group, were obtained by BLAST runs against the nr database with the peroxisomal mouse (BAB23628.1) and the *Pseudomonas stutzeri* (WP 017245866.1) sequences as query. The multiple sequence alignments of the TFEα1 and TFEα2 sequences, including the trypanosomal and leishmanial orthologous sequences found in TriTrypDB (http://tritrypdb.org/tritrypdb/) were done with the web-based CLUSTALW2 program, and the guide tree obtained was used to construct a dendrogram using the TreeView program.

### Knockout of the TFE candidate gene

Replacement of the putative enoyl-CoA hydratase/enoyl-CoA isomerase/3-hydroxyacyl-CoA dehydrogenase (*TFE*, Tb927.2.4130) by the puromycin (*PAC*) and blasticidin (*BSD*) resistance markers *via* homologous recombination was performed with DNA fragments containing a resistance marker gene flanked by the *TFEα1* UTR sequences. The *TFEα1* knock out was generated in the EATRO1125.T7T parental cell line, which constitutively expresses the T7 RNA polymerase gene and the tetracycline repressor under the control of a T7 RNA polymerase promoter for tetracycline inducible expression (TetR-HYG T7RNAPOL-NEO) [21]. Transfection and selection of drug-resistant clones were performed as previously reported [30]. The first and second *TFEα1* alleles were replaced by *PAC* and *BSD*, respectively. Transfected cells were selected in SDM79 medium containing hygromycin B (25 µg/ml), neomycin (10 µg/ml), puromycin (1 µg/ml) and blasticidin (10 µg/ml). The selected cell line (TetR HYG T7RNAPOL NEO Δ*tfeα1*::PAC/Δ*tfeα1*::BSD) is abbreviated as Δ*tfeα1*/Δ*tfeα1*.

### Southern blot analysis

6 µg of genomic DNA from the wild-type and Δ*tfeα1*/Δ*tfeα1* cell lines, extracted as previously described [31], were digested with the KpnI restriction enzyme, separated by electrophoresis in a 0.8% agarose gel and transferred onto a nylon membrane (Hybond N^+^, Roche Molecular Biochemicals). The membrane was hybridized with digoxigenin-labeled DNA probes synthesized with a PCR DIG probe synthesis kit (Roche Molecular Biochemicals) as recommended by the supplier. The *TFEα1* and *FRD* probes were generated by PCR amplification, using the primer pairs 5′-ATGCGTCGCTTGGAAACCATATC-3′/5′-GAGCCGCTGCTGCTGTAGTCCCG-3′ and 5′-GTGTAACGTCGTTGCTCAGTGAGA-3′/5′-GCGAAATTAAATGGGCCCCGC GACG-3′, respectively. Probe-target hybrids were visualized by a chemiluminescent assay with the DIG luminescent detection kit (Roche Molecular Biochemicals), according to the manufacturer’s instructions. Blots were exposed to ImageQuant LAS4010 (*GE* Healthcare Life Sciences) for approximately 20 min.

### Sample preparation for proteomic analysis

Glycosome enriched fractions were loaded on a 10% acrylamide SDS-PAGE gel. Migration was stopped when samples had just entered the resolving gel, proteins were visualized by Colloidal Blue staining, and the unresolved region of the gel cut into 1 mm×1 mm gel pieces. Gel pieces were destained in 25 mM ammonium bicarbonate (NH_4_HCO_3_), 50% Acetonitrile (ACN) and shrunk in ACN for 10 min. After ACN removal, gel pieces were dried at room temperature. Proteins were first reduced in 10 mM dithiothreitol, 100 mM NH_4_HCO_3_ for 30 min at 56°C then alkylated in 100 mM iodoacetamide, 100 mM NH_4_HCO_3_ for 30 min at room temperature and shrunken in ACN for 10 min. After ACN removal, gel pieces were rehydrated with 100 mM NH_4_HCO_3_ for 10 min at room temperature. Before protein digestion, gel pieces were shrunken in ACN for 10 min and dried at room temperature. Proteins were digested by incubating each gel slice with 10 ng/µl of trypsin (T6567, Sigma-Aldrich) in 40 mM NH_4_HCO_3_, 10% ACN, rehydrated at 4°C for 10 min, and finally incubated overnight at 37°C. The resulting peptides were extracted from the gel by three steps: a first incubation in 40 mM NH_4_HCO_3_, 10% ACN for 15 min at room temperature and two incubations in 47.5% ACN, 5% formic acid for 15 min at room temperature. The three collected extractions were pooled with the initial digestion supernatant, dried in a SpeedVac, and resuspended with 25 µl of 0.1% formic acid before nanoLC-MS/MS analysis.

### NanoLC-MS/MS analysis

Online nanoLC-MS/MS analyses were performed using an Ultimate 3000 system (Dionex, Amsterdam, The Netherlands) coupled to a nanospray LTQ Orbitrap XL mass spectrometer (Thermo Fisher Scientific, Bremen, Germany). Ten microliters of each peptide extract were loaded on a 300 µm ID×5 mm PepMap C_18_ precolumn (LC Packings, Dionex, USA) at a flow rate of 20 µl/min. After 5 min desalting, peptides were online separated on a 75 µm ID×15 cm C_18_PepMap™ column (LC packings, Dionex, USA) with a 2–40% linear gradient of solvent B (0.1% formic acid in 80% ACN) during 108 min. The separation flow rate was set at 200 nl/min. The mass spectrometer operated in positive ion mode at a 1.8 kV needle voltage and a 42 V capillary voltage. Data were acquired in a data-dependent mode alternating an FTMS scan survey over the range m/z 300–1700 with the resolution set to a value of 60 000 at m/z 400 and six ion trap MS/MS scans with Collision Induced Dissociation (CID) as activation mode. MS/MS spectra were acquired using a 3 m/z unit ion isolation window and normalized collision energy of 35. Mono-charged ions and unassigned charge-state ions were rejected from fragmentation. Dynamic exclusion duration was set to 30 sec.

### Database search and results processing

Mascot and Sequest algorithms through Proteome Discoverer 1.4 Software (Thermo Fisher Scientific Inc.) were used for protein identification in batch mode by searching the *Trypanosoma brucei* TREU927 database (TritrypDB release 6.0, 90307 entries) at http://tritrypdb.org/. Two missed enzyme cleavages were allowed. Mass tolerances in MS and MS/MS were set to 10 ppm and 0.6 Da. Oxidation of methionine, acetylation of lysine and deamidation of asparagine and glutamine were searched as variable modifications. Carbamidomethylation on cysteine was searched as fixed modification. Peptide validation was performed using Percolator algorithm [32] and only “high confidence” peptides were retained corresponding to a 1% false positive rate at peptide level.

### Label-Free Quantitative Data Analysis

Raw LC-MS/MS data were imported in Progenesis LC-MS 4.1 (Nonlinear Dynamics Ltd, Newcastle, U.K) for feature detection, alignment, and quantification. All sample features were aligned according to retention times by manually inserting up to two hundred landmarks followed by automatic alignment to maximally overlay all the two-dimensional (m/z and retention time) feature maps. Singly charged ions and ions with higher charge states than six were excluded from analysis. All remaining features were used to calculate a normalization factor for each sample that corrects for experimental variation. Peptide identifications (with p<0.01, see above) were imported into Progenesis. For quantification, all unique peptides of an identified protein were included and the total cumulative abundance was calculated by summing the abundances of all peptides allocated to the respective protein. No minimal thresholds were set for the method of peak picking or selection of data to use for quantification. For each biological replicate, the mean normalized intensities and standard deviation were calculated and ratio was deducted. Noticeably, only non-conflicting features and unique peptides were considered for calculation at protein level. Quantitative data were considered for proteins quantified by a minimum of 2 peptides. As an indication of the confidence of that protein’s presence, the sum of the peptide scores (confidence score) is calculated for each protein from the search algorithm. This score includes unique peptides as well as switched off peptides, the later decreasing the confidence score.

## Results

We started with the hypothesis that carbon storage in the form of lipid droplets (LD) is a physiological adaptation to nutrient supply in *T. brucei* and quantified LDs under excess fatty acid feeding conditions. Oleate was chosen as fatty acid species previously shown to be taken up and metabolized by trypanosomes [15]. The lipid or phospholipid content of bloodstream and procyclic *T. brucei* cells has been determined [33], [34], but no detailed analyses of the TAG species has been reported. We investigated procyclic trypanosomes, as lipid storage may be advantageous to face the nutritional bottlenecks during their subsequent development in the tsetse.

### Oleate uptake and storage in lipid droplets

**Figure 1:**
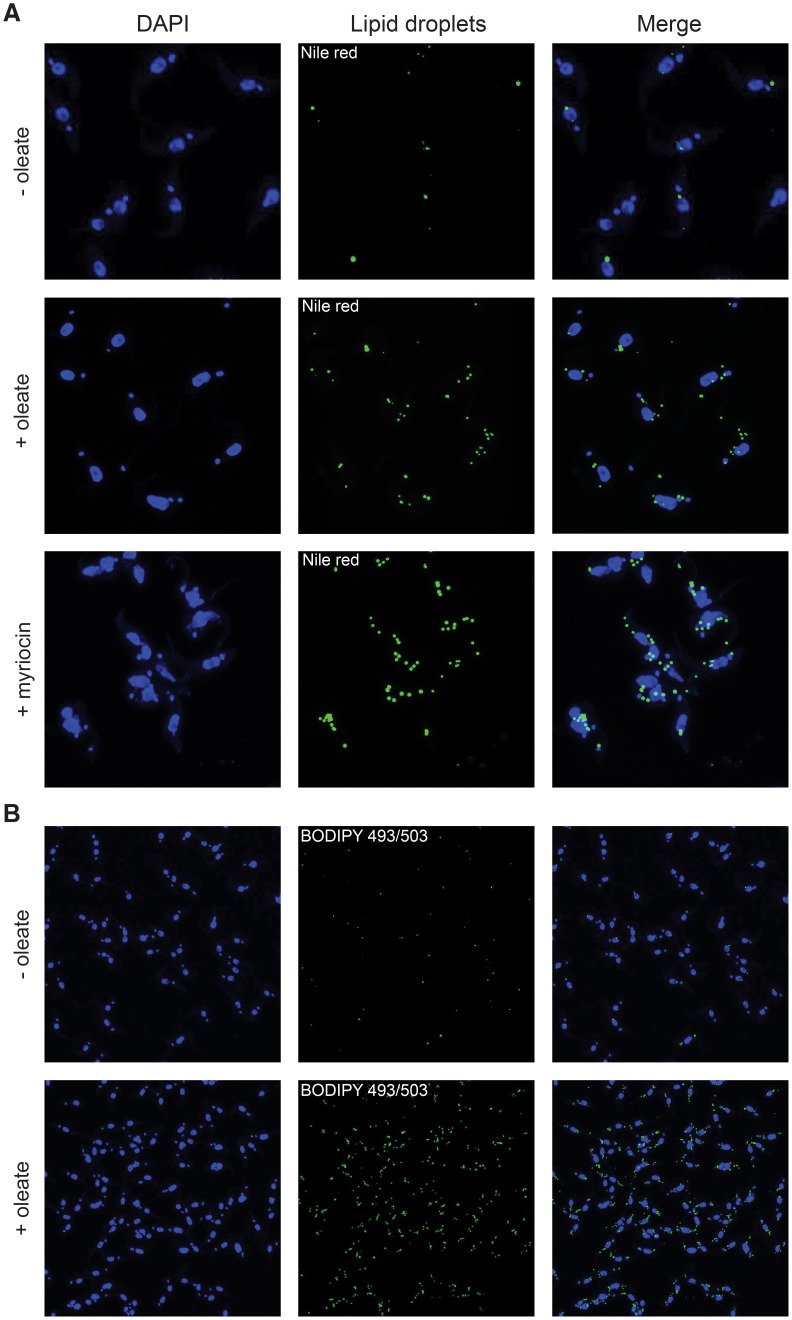
Oleate feeding stimulates lipid droplet formation in procyclic *T. brucei* cells. Staining of lipid droplets with nile red (A) or BODIPY 493/503 (B) was as detailed in experimental procedures. Myriocin treatment (0.5 µM for 24 h) was included for comparison to a previous report [36]. An example of several experiments is shown.

**Figure 2:**
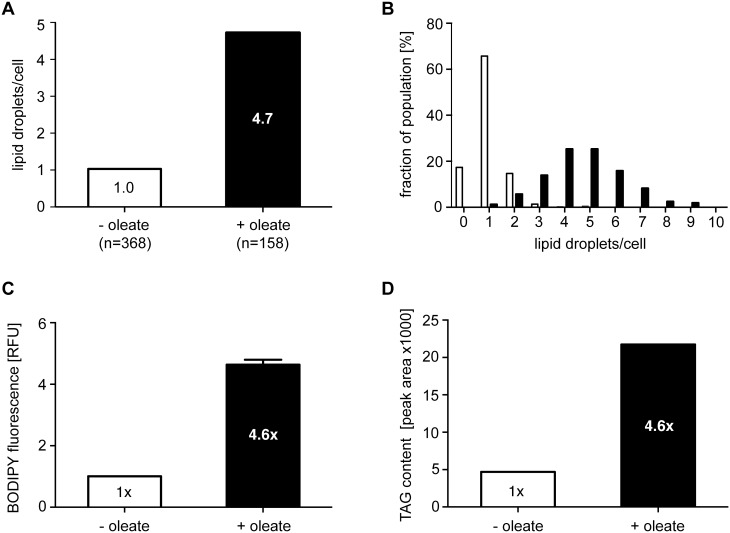
Quantification of the oleate-induced lipid droplet formation. (A) BODIPY 493/503 stained LDs were counted in stacks of confocal laser scanning microscopy (CLSM) images; the average number of LDs per cell is given after oleate feeding (black column) or in the control (white column). (B) Distribution of LD numbers per cells in the population after oleate feeding (black columns) or in the control (white columns). (C) Quantification of BODIPY-stained LDs by flow cytometry after oleate feeding (black column) or in the control (white column). BODIPY 493/503 preferentially stains nonpolar lipids. Error bars give the SEM (n = 3) of values normalized to the control. (D) Quantification of TAG content by HPTLC and densitometry after oleate feeding (black columns) or in the control (white columns). Values are normalized to the control.

**Figure 3:**
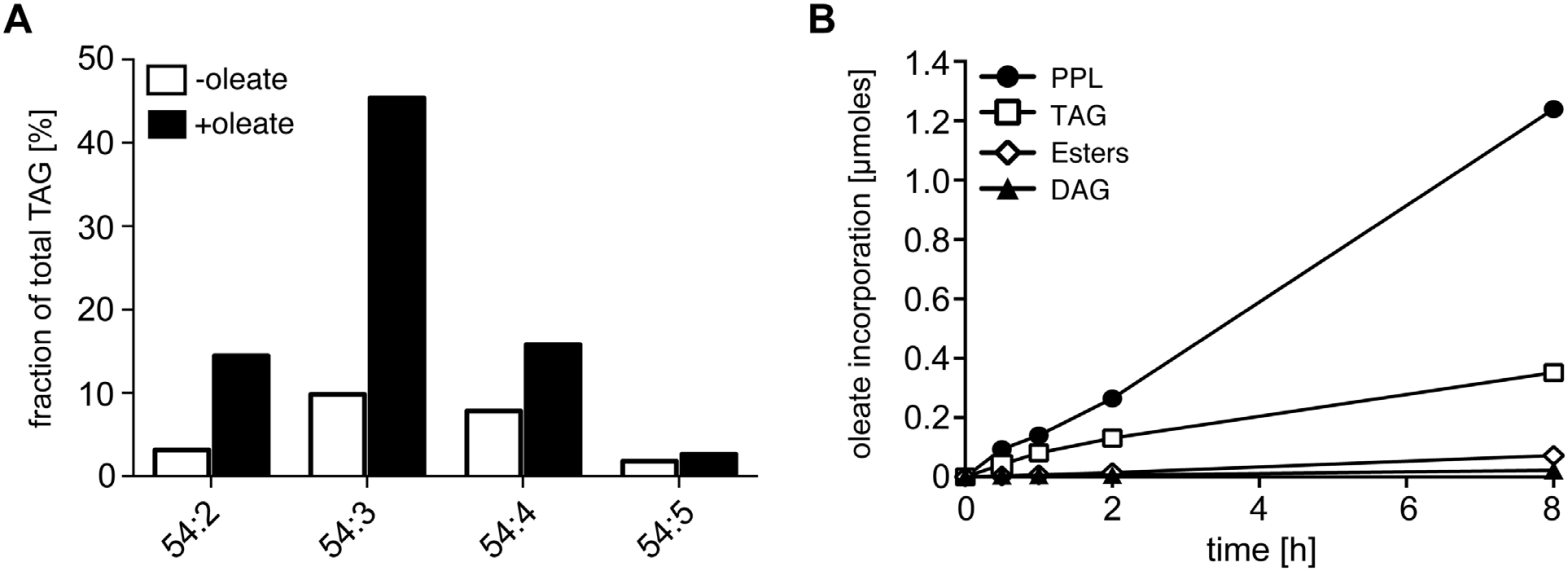
TAG species analysis and uptake of labeled oleate. (A) Dominant TAG species in procyclic *T. brucei* cells identified by ESI/MS/MS after oleate feeding for three days (black columns) or in the control (white columns). For a complete list of TAG species detected see S1 Figure. The nomenclature 54:X indicates the total carbon number of all three acyl chains and the sum of all unsaturated double bonds within the acyl chains. (B) Uptake kinetics upon growth in the presence of radiolabeled oleate for up to 8 h. The incorporation of ^14^C oleate into lipid species was quantified by HPTLC and a Storm 860 phosphorimager. PPL, phospholipids; TAG, triacylglycerol; SE, Steryl-esters; DAG, diacylglycerol.

After oleate/BSA feeding of procyclic trypanosomes for 2–3 days the number of nile red stained LDs increased (Fig. 1A), as previously shown upon drug treatment with myriocin [35] and included here for reference (Fig. 1A). Whereas myriocin treatment led to a cytokinesis phenotype [35], feeding with oleate/BSA did not change the growth rate. The effect of oleate feeding was quantified by counting the number of nile red stained LDs per cell in stacks of confocal laser scanning images. The average number of LDs per cell increased almost 5-fold compared to unfed cells (Fig. 2A). The histogram in Fig. 2B shows the bell-shaped, apparently normal, distribution of the LD numbers per cell in the populations. The maximum number of LDs that a single cell can build up, nine LDs in oleate fed cells in our experiments with strain AnTat1.1, may depend on cell clone-specific properties like uptake capacity and growth rate. A similar argument applies to the average number of lipid droplets in unfed cells that is also likely to depend on the batch of FCS and the amount of fatty acids (FAs) contained within. As a routine assay to quantify LDs in *T. brucei*, we optimized flow cytometry after BODIPY 493/503 staining. The microscopic picture upon BODIPY 493/503 staining is not different from nile red staining (Fig. 1B). Yet, nile red has wide and overlapping emission spectra when bound to polar and nonpolar lipids, whereas BODIPY 493/503 accumulates more specifically in the nonpolar lipophilic environment in LDs [20]. Flow cytometry integrates the fluorescence signal of the whole cell, and therefore low background from membrane lipid staining is essential for LD quantification by flow cytometry. The validity of the flow cytometric assay was demonstrated by an increase of the fluorescence signal between the unfed and oleate fed cells (Fig. 2C), that was very close (4.6-fold) to the increase determined by microscopic LD counting (4.7-fold, Fig. 2A). The TAG content of cells incubated with or without oleate was also directly quantified by thin layer chromatography (TLC) (Fig. 2D), again resulting in the very same increase (4.6-fold). The perfect quantitative correlation of LD numbers, flow cytometry and TAG analysis upon oleate feeding, strongly suggests that oleate uptake results in TAG storage in LDs. The TAG species in oleate fed and unfed cells were then analyzed by mass spectrometry. A high number of 96 TAG species were resolved and identified (S1 Figure). Such a high number of TAG species has already been observed in serum and butter [37], [38]. In both conditions the 54∶2,3,4 TAG species were by far the predominant species and were significantly increased upon oleate feeding (Fig. 3A). As oleate is a C18 fatty acid with one unsaturated double bond, the predominant 54∶3 TAG species provides evidence that at least part of the oleate taken up is esterified with glycerol for storage in lipid droplets. To directly follow incorporation of oleate into TAGs, we performed a labeling experiment with [^14^C]-oleate (Fig. 3B). Procyclic trypanosomes were cultured in the presence of radiolabeled oleate up to 8 hours. Samples were collected during this uptake time course and labeled lipid species were separated by TLC and quantified using a phosphor imager. Oleate was incorporated into TAG as well as into phospholipids (PPL) in a time-dependent manner.

### Characterisation of a β-oxidation pathway candidate gene

**Figure 4:**
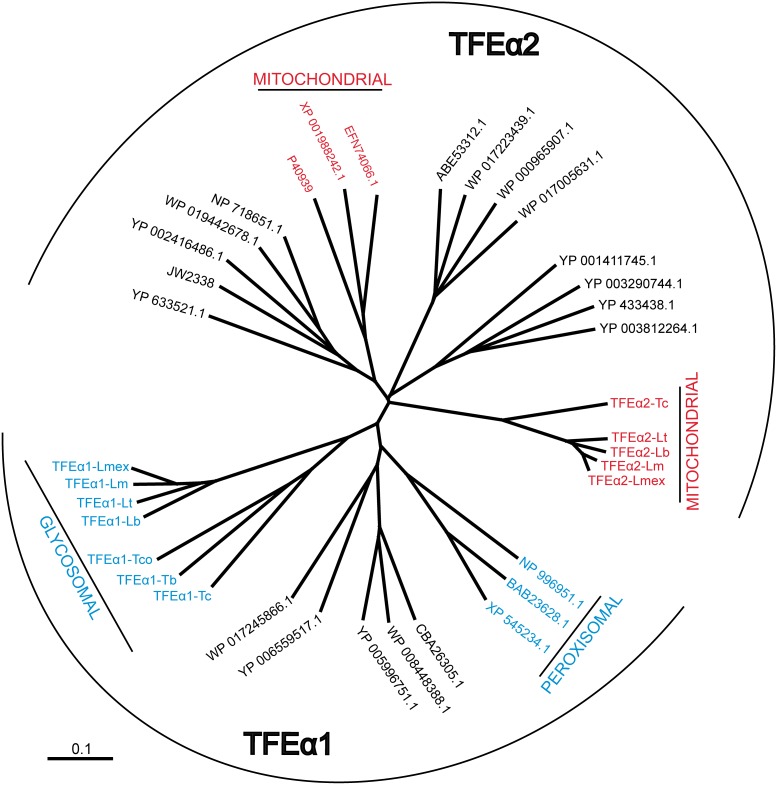
Dendrogram of trifunctional enzyme (TFE) isoforms. Prokaryotic (black characters) and eukaryotic (colored characters) TFEα sequences are represented by their GenBank accession codes. Glycosomal/peroxisomal (TFEα1) or mitochondrial (TFEα2) proteins are highlighted in blue and red. Experimental evidence for glycosomal localization of trypanosomatid TFEα1 isoforms, which all contain a PTS2 motif, is limited to *T. brucei* TFEα1 (see [40] and S4 Figure). Mitochondrial localization of the trypanosomatid TFEα2 isoforms is assumed due to an N-terminal mitochondrial targeting motif and the absence of a PTS motif. Abbreviations: Lb, *Leishmania braziliensis*; Lm, *L. major*; Lmex, *L. mexicana*; Lt, *L. tarentolae*; Tb, *T. brucei*; Tc, *T. cruzi*; Tco, *T. congolense*. The organisms corresponding to the accession numbers are: *Canis lupus familiaris* (XP_545234.1), *Danio rerio* (NP_996951.1), *Mus musculus* (BAB23628.1), *Curvibacter* putative symbiont of *Hydra magnipapillata* (CBA26305.1), *Janthinobacterium* sp. HH01 (WP_008448388.1), *Marinobacter* sp. BSs20148 (YP_006559517.1), *Pseudomonas stutzeri* (WP_017245866.1), *Ralstonia solanacearum* CMR15 (YP_005996751.1), *Camponotus floridanus* (EFN74066.1), *Drosophila grimshawi* (XP_001988242.1), *γ-proteobacterium* HdN1 (YP_003812264.1), *Hahella chejuensis* KCTC2396 (YP_433438.1), *Homo sapiens* (P40939), *Moritella dasanensis* (WP_017223439.1), *Parvibaculum lavamentivorans* DS-1 (YP_001411745.1), *Rhodothermus marinus* DSM4252 (YP_003290744.1), *Shewanella denitrificans* OS217 (ABE53312.1), *Vibrio splendidus* LGP32 (YP_002416486.1), *Escherichia coli* (JW2338), *Enterovibrio norvegicus* (WP_017005631.1), *Moritella marina* (WP_019442678.1), *Myxococcus xanthus* DK1622 (YP_633521.1), *Shigella flexneri* (WP_000965907.1), *Shewanella oneidensis* MR-1 (NP_718651.1).

**Figure 5:**
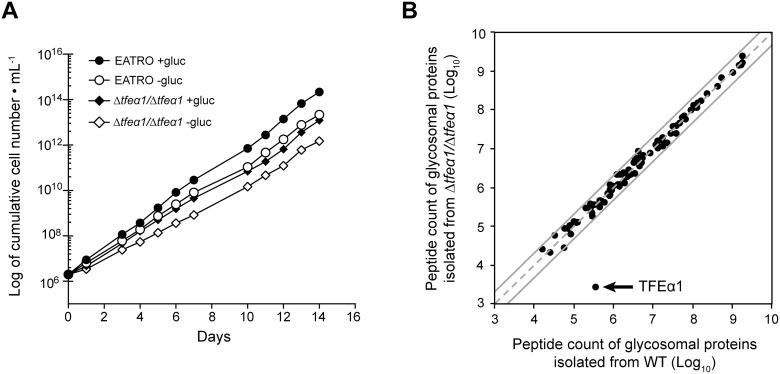
Phenotypic analysis of Δ*tfeα1*/Δ*tfeα1* cell. (A) growth curve of WT and Δ*tfeα1*/Δ*tfeα1* cell knock cells in glucose-rich (SDM79 with 10 mM glucose) or glucose-free (SDM79GluFree) conditions. (B) Global protein abundance in the partially purified glycosome fraction of WT (x-axis) and Δ*tfeα1*/Δ*tfeα1* cell knock cells (y-axis). Each protein identification is presented by a point at log_10_ of normalized peptide count values taken from the proteome data in S4 Figure. Proteins on the dashed grey line have identical normalized peptide counts in both samples; the grey lines represent a 2-fold abundance in one condition.

A likely rationale for uptake and storage of lipids in a specific cellular compartment is later use for energy production by β-oxidation. In cell lysates of procyclic *T. brucei* the enzymatic activities of 2-enoyl-CoA hydratase and 3-hydroxyacyl-CoA dehydrogenase, two essential enzymatic steps in β-oxidation have previously been detected [9]. In order to explore the genomic capacity for β-oxidation in *T. brucei*, a bioinformatic search for candidate genes for these two activities was undertaken. The β-oxidation pathway consists of four steps, being an acyl-CoA dehydrogenation, an enoyl-CoA hydratation, a 3-hydroxyacyl-CoA dehydrogenation and a thiolytic cleavage reaction. In most organisms, the first reaction of this pathway is catalyzed by a monofunctional enzyme, while the three other reactions are catalyzed by a trifunctional enzyme (TFE) complex, composed of a bifunctional TFEα subunit (enoyl-CoA hydratase and 3-hydroxyacyl-CoA dehydrogenase activities) and a monofunctional TFEβ subunit (thiolase activity). Most eukaryotes contain two phylogenetically distinct TFEα, one located in the mitochondrion (named TFEα2) and the other in peroxisomes (named TFEα1). The *Leishmania spp.* and *T. cruzi* genomes contain one mitochondrial and one glycosomal type gene with a mitochondrial targeting motif or a peroxisomal targeting sequence 2 (PTS2) present, respectively. However, only one gene encoding the putative glycosomal TFEα1 isoform, is detected in the African trypanosome genomes (Fig. 4). We have searched by BLAST not only the Tb427 genome but also the Tb927, *T. gambiense* and *T. congolense* genomes in TritrypDB and in addition our unpublished AnTat1.1 genome assembly. There is no trace of a second *TFEα*-like gene in salivarian trypanosomes. This leaves *T. brucei* with a single candidate gene for the measured enoyl-CoA hydratase and 3-hydroxyacyl-CoA dehydrogenase activities. Therefore, the *TFEα1* candidate gene was deleted by a homologous recombination-mediated homozygous gene replacement with two antibiotic resistance markers. The identity of the resulting Δ*tfeα1*/Δ*tfeα1* null mutant was verified by locus PCR and by Southern blot analysis (S3 Figure). As glucose starvation may induce the putative β-oxidation pathway to restore the energy balance, the growth rate of WT and Δ*tfeα1*/Δ*tfeα1* null mutant cells was determined in our new glucose-free medium (SDM79GluFree, see Methods) supplemented or not with 10 mM glucose. Growth of the null mutant is only moderately affected compared to WT regardless of the amount of glucose (Fig. 5A). TFEα1 contains a peroxisomal targeting signal 2 motif (PTS2, RLETISSHV) [38] and has recently been found enriched in glycosomal fractions [39]. In addition, TFEα1 contains a putative 24 amino acid N-terminal mitochondrial target motif predicted by MitoProt (http://ihg.gsf.de/ihg/mitoprot.html) with a moderate probability (0.41). In absence of antibody reagents, we used proteomic analysis of glycosome enriched fractions from WT and Δ*tfeα1*/Δ*tfeα1* null mutant cells to probe expression and subcellular localization. We compared the ratio of peptide counts of WT over Δ*tfeα1*/Δ*tfeα1* for all glycosomal proteins that Güther et al. [39] detected with confidence in their proteome of affinity purified glycosomes (Fig. 5B, S4 Figure). A ratio around 1 for all proteins detected, showed that the protein composition of glycosomes is not altered in the Δ*tfeα1*/Δ*tfeα1* mutant cells. Only for TFEα1, a 140-fold ratio of peptide counts of WT over Δ*tfeα1*/Δ*tfeα1* was detected and demonstrated that the candidate gene product is expressed in procyclic *T. brucei.* Enzymatic activity was then measured in WT and Δ*tfeα1/*Δ*tfeα1* knockout cells using whole cell extracts and partially purified glycosome fractions. Only NADPH-dependent 3-hydroxyacyl-CoA dehydrogenase activity was detected with C4 substrate (Table 1), but no NADH-dependent activity (not shown). When considering the different cell fractionation methods, our values for NADPH-dependent 3-hydroxyacyl-CoA dehydrogenase activity are consistent with those previously reported in [9]. Surprisingly, the activity was not significantly different in WT and Δ*tfeα1/*Δ*tfeα1* whole cell lysates and in the respective glycosome preparations. We conclude that the *TFEα1* candidate gene cannot encode NADPH-dependent 3-hydroxyacyl-CoA dehydrogenase activity. A bona fide glycosomal activity, glycerol-3-phosphate dehydrogenase (GPDH), is 7-fold enriched in our partially purified glycosome preparations, while the NADPH-dependent 3-hydroxyacyl-CoA dehydrogenase activity is less than 2-fold enriched (Table 1), which is consistent with previous localization of the latter activity in several subcellular compartments [9]. We cannot formally exclude that the *TFEα1* candidate gene encodes a distinct 3-hydroxyacyl-CoA dehydrogenase enzyme that is completely inactive in procyclic trypanosomes. However, the NADPH-dependent 3-hydroxyacyl-CoA dehydrogenase activity reported here and in [9] is clearly not encoded by *TFEα1*. As the putative β-oxidation pathway may be induced by glucose starvation, we measured the 3-hydroxyacyl-CoA dehydrogenase activity in both WT and Δ*tfeα1*/Δ*tfeα1* cells grown in SDM79GluFree for one week, but no differences were observed compared to glucose-rich conditions. In summary, previous arguments in favor of a β-oxidation pathway in *T. brucei* now rely on the NADP-dependent and possibly anabolic activities reported in [9], whereas no metabolic function can be detected so far for the annotated *TFEα1* candidate gene [38].

**Table 1:** NADPH-dependent 3-hydroxyacyl-CoA dehydrogenase activity in WT and Δ*tfeα1*/Δ*tfeα1* cells.

	3 hydroxyacyl-CoA	GPDH^3^
	dehydrogenase^3^	
WT WCE^1^+gluc^4^	6.62±0.63 (n = 5)	32.20±3.48 (n = 3)
WT WCE^1^−gluc^5^	5.22±0.40 (n = 5)	34.92±2.71 (n = 3)
Δ*tfeα1/*Δ*tfeα1* WCE^1^+gluc^4^	6.04±0.71 (n = 5)	22.80±2.45 (n = 3)
Δ*tfeα1/*Δ*tfeα1* WCE^1^−gluc^5^	5.00±0.47 (n = 5)	35.40±1.89 (n = 3)
WT glyco^2^+gluc^4^	11.76±0.52 (n = 6)	213.18±4.12 (n = 3)
Δ*tfeα1/*Δ*tfeα1* glyco^2^+gluc^4^	9.12±0.77 (n = 6)	208.22±12.19 (n = 3)

1 WCE, whole cell exctract.

2 glyco, partially purified glycosome fraction.

3 Mean ± SEM of n experiments (mU/mg of protein).

4 +gluc: cells cultured in SDM79 containing 10 mM glucose.

5 −gluc: cells cultured in glucose-depleted SDM79GluFree.

### Lipid droplet and TAG turnover

**Figure 6:**
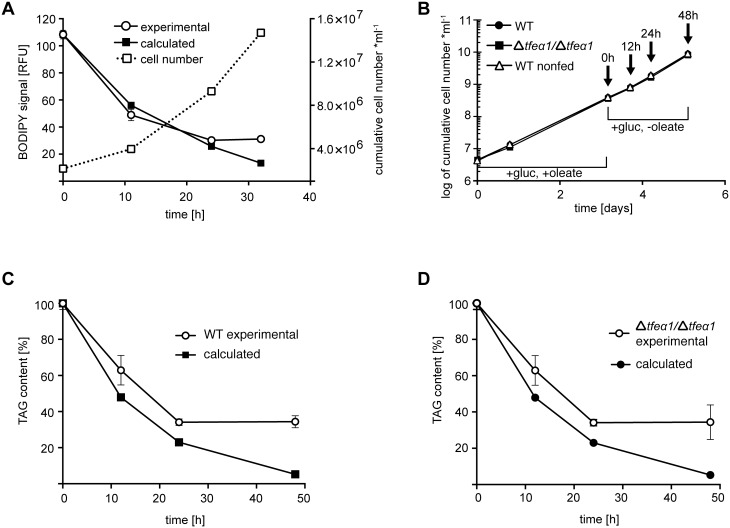
LD and TAG turnover in WT and Δ*tfeα1*/Δ*tfeα1* cells. Cells were fed with oleate in glucose-rich SDM79 medium for three days, and after oleate withdrawal samples were taken at the time points indicated. (A) WT cells stained with BODIPY and analyzed by flow cytometry (left y-axis). Error bars represent the SEM of independent replicates (n = 3). The growth curve is given as dashed line (right y-axis). (B) Growth curve and sampling time points (arrows) for the experiments in panels (C) and (D). Total TAG content was determined in triplicate by HPTLC and densitometry in WT (C) and Δ*tfeα1*/Δ*tfeα1* (D) cells. Error bars represent the SEM of independent replicates (n = 3). The calculated values (filled symbols) account for dilution of LDs or TAG content by cell division, based on the matched growth data.

The fate of the accumulated LDs in oleate fed cells was then determined. We quantified the kinetics of LD decay upon oleate withdrawal and culture in normal SDM79 medium. The LD decay kinetic was first analyzed by flow cytometry with BODIPY 493/503 staining. After maximal feeding for 3 days, samples were collected over a period of 32 hours. We assumed that in a growing cell population the preformed lipid droplets are equally distributed to daughter cells and therefore calculated the expected fluorescent signal decrease using the population doubling time in the actual experiment, as derived from the growth curve in Fig. 6A. The thereby calculated decay kinetics is represented by filled squares in Fig. 6A. The fluorescence decrease measured from flow cytometry data (open circles) was identical with the calculated kinetic until a basal level was reached. Thus, dilution during cell divisions can fully account for the initial kinetics of LD decay down to basal level. The same kinetic experiment was performed with quantification of the total TAG content by TLC. The growth curve and sampling time points are shown in Fig. 6B and the TAG content kinetics in Fig. 6C, D. Again, a very similar decrease of calculated and experimentally determined TAG content is seen upon oleate withdrawal. Whereas the calculated dilution curve predicts very low TAG levels after several cell cycles, the experimental values return to the basal level maintained by the lipid uptake in normal medium and lipid synthesis. Importantly, the experimental values were never found below the calculated prediction. In summary, there is no net catabolism of the accumulated and stored TAGs, which does not however exclude balanced rates of lipid uptake and degradation in steady state conditions. The Δ*tfeα1/*Δ*tfeα1* null mutant was also analyzed in this experiment (Fig. 6D). The results were identical, and the kinetics for WT and Δ*tfeα1/*Δ*tfeα1* were perfectly superimposed. This was expected if TFEα1 was not involved in lipid catabolism in procyclic trypanosomes.

## Discussion

Carbon storage is widespread in organisms to maintain energy homeostasis during transient nutrient shortage and periods of starvation of individual cells or of metazoan organisms. The predominant forms of storage carbon are fat in the form of triacylglycerol and carbohydrate polymers like glycogen in animals and yeast or starch in plants [40]–[42]. In the kinetoplastid protozoan *Leishmania major* the carbohydrate polymer mannan has apparently replaced glycogen [43]. In *Trypanosoma* mannan has not been detected, but lipid droplets (LDs) have been described as a regulated compartment [8], compatible with a role in lipid storage. LD biogenesis is dependent on a protein kinase, termed LDK (lipid droplet kinase) as shown by RNAi-mediated repression [8]. An electron microscopic study reports that number and size of LDs vary during insect stage differentiation from the midgut to the salivary glands [14]. These observations are correlative, but point to a physiological function in the parasités adaptation, probably to nutritional bottlenecks during development and migration in the tsetse alimentary tract. Here we report for the first time that an induced physiological change in environmental conditions, namely the supplementation of cell culture medium with fatty acids (oleate), can stimulate the buildup of LDs in procyclic *T. brucei* without any impact on the celĺs growth rate. The inhibitor myriocin also increased LD numbers in procyclic *T. brucei* in a previous report, but caused a severe cytokinesis phenotype [8]. We provide evidence that oleate is taken up and esterified to triacylglycerol (TAG) for storage in LDs: (1) upon feeding, the LD number, the quantity of stained lipids and the cellular TAG content increase by the very same factor of 4–5; (2) radiolabelled oleate is incorporated into TAGs (and phospholipids); (3) out of 96 TAG species detected by mass spectrometry, the 54∶3 TAG species (e.g. oleate) was by far the most abundant in cells fed and unfed with oleate. The fact that in unfed cells the most abundant TAG species was of the 54∶3 type, suggests that oleate (18∶1) is preferentially used for storage in trypanosomes.

The question remains how these lipid stores are used by the cell. One possibility is their use for rapid synthesis or remodeling of membrane lipids upon proliferation or differentiation under limiting nutrient supply. In *T. cruzi* for example, the fatty acid composition of phospholipids (PPL) changes in response to the environmental temperature. Increasing temperature causes a higher ratio of saturated to unsaturated fatty acids in PPL, this being balanced by an inverse change in cellular TAG pools, that may represent LDs. Exchange of fatty acids between the TAG pool and the membrane PPL pool maybe part of an environmental adaptation [44]. The alternative fate of lipid stores is catabolism for energy production upon starvation. We first considered the most widespread pathway of fatty acid catabolism present in most organisms, fatty acid β-oxidation. This was motivated by the previous report of enzymatic activities compatible with a β-oxidation pathway in *T. brucei* [9] and the prediction of a candidate gene in the genome [38]. In contrast to expectation, the only recognizable candidate gene, *TFEα1,* did not encode the reported activity, in spite of evidence for TFEα1 expression. This has significantly weakened previous arguments in favor of glycosomal β-oxidation in *T. brucei.* The reported 3-hydroxyacyl-CoA dehydrogenase activity was also independent of the presence or absence of glucose (Table 1). The gene encoding this activity is not known and it remains possible that the relatively low activity in crude lysates is a side activity of an enzyme not involved in β-oxidation. Another previous argument for catabolism by β-oxidation was the identification of a glycosomal ABC transporter (GAT1) with a specificity for oleoyl-CoA, which becomes essential in the absence of glucose [45]. However, this transporter might also be important to supply ether lipid biosynthesis in glycosomes [46], [47]. The kinetics of LD decay and decrease of cellular TAG content upon oleate withdrawal (Fig. 6) can be fully accounted for by the dilution effect of cellular proliferation. Thus, there is apparently no net catabolism of lipids stored in LDs in procyclic trypanosomes under those conditions. This contrasts with *Leishmania spp.* that can take up fatty acids in culture, with evidence for esterification and catabolism by β-oxidation [48]–[50]. β-oxidation was also reported for *T. cruzi* [51] and *T. gondii* [52], [53], and in *C. fasciculata* α-oxidation has been shown [54], suggesting subsequent β-oxidation. Interestingly, the lack of experimental evidence for β-oxidation in *T. brucei* correlates with the presence in the African trypanosome genomes of only one gene encoding a putative TFEα1 subunit of the trifunctional enzyme complex. The *Leishmania spp.* and *T. cruzi* genomes contain two *TFEα1* candidates, one mitochondrial and one glycosomal type gene with a mitochondrial targeting motif or a peroxisomal targeting sequence 2 (PTS2), respectively. It is convincible that a functional pathway has been lost during evolution of *Trypanosomatidae.* Alternatively, TFEα1 in *T. brucei* may be an enzyme activated only in a developmental stage in the tsetse, that is not available for biochemical analysis. The absence of a growth phenotype in Δ*tfeα1/*Δ*tfeα1* knockout cells and the unchanged glycosomal proteome in these mutant cells are compatible with a strictly developmental stage-specific function. Also, in cultured *L. major,* significant β-oxidation flux or a physiological role of that pathway have not been detected, and the contribution of fatty acids to TCA cycle intermediates was rather minor compared to the contribution of amino acids [55]. This opens the possibility, that also in *Leishmania* the pathway may be activated only in developmental stages not investigated in that study.

Our study shows that available fatty acids can be stored as TAG in lipid droplets, but the developmental stages using those stores and the pathways involved remain to be investigated.

## Supporting Information

S1 Figure **TAG species identified in procyclic** ***T. brucei*** **cells.** Relative abundances of TAG species were determined by ESI/MS/MS after oleate feeding for three days (black columns) or in the control (white columns). The nomenclature 54:X indicates the total carbon number of all three acyl chains and the sum of all unsaturated double bonds within the acyl chains. Most TAG species are minor contributions to the total TAG content.
(PDF)

S2 Figure **Alignment of TFEα1 and TFEα2 protein sequences.** The TriTrypDB IDs (http://tritrypdb.org/tritrypdb/) of trypanosomatid sequences are LmjF.33.2600 (TFE1-Lm), LmxM.32.2600 (TFE1-Lmex), LbrM.33.2880 (TFE1-Lb), LtaP33.2830 (TFE1-Lt), Tb927.2.4130 (TFE1-Tb), TcIL3000_2_640 (TFE1-Tco), TcCLB.507547.40 (TFE1-TcCLB), LmjF.26.1550 (TFE2-Lm), LmxM.26.1550 (TFE2-Lmex), LbrM.26.1570 (TFE2-Lb), LtaP26.1590 (TFE2-Lt), TcCLB.508981.39 (TFE2-TcCLB). The names of TFEs from other species correspond to their GenBank accession numbers. Gaps (-) were introduced to maximize the alignments. The graphical output of the Clustal alignment was performed with CLC Main Workbench 6.
(PDF)

S3 Figure **Verification of the Δ** ***tfeα1*** **/Δ** ***tfeα1*** **null mutant.** (A) Verification of the Δ*tfeα1*/Δ*tfeα1* null mutant by integration control PCRs. The lanes of the gel are numbered according to the primer combinations used. (B) Southern blot analysis of the Δ*tfeα1*/Δ*tfeα1* null mutant. Hybridization of KpnI-digested wild-type genomic DNA with the TFE*α*1 probe revealed the expected 2.2 kb band, whereas loss of this band in the Δ*tfeα1*/Δ*tfeα1* genomic DNA is diagnostic for loss of the *TFEα1* gene. As a control, hybridization of the same blot with the fumarate reductase (FRD) probe showed the identical band pattern in both wild-type and Δ*tfeα1*/Δ*tfeα1* cell lines, corresponding to the FRDg and FRDm2 genes. DNA fragment sizes are indicated in kilobases (kb).
(PDF)

S4 Figure **Proteomic analysis of a glycosomal preparation.** From proteome analysis of the glycosome enriched fraction from WT and Δ*tfeα1*/Δ*tfeα1* cell lines, only the glycosomal enzymes, identified with high confidence (group III) in Güther et al [40] are shown in this table. The TriTrypDB ID and name of each protein is indicated in the 1^st^ and 2^nd^ columns. The data represent the SEM of 3 analyses of the same samples (technical replicate). Log_10_ of the “mean” values are used in Figure 5B. The last column indicates the ratio between the WT and Δ*tfeα1*/Δ*tfeα1* data.
(XLS)
