# Supplementary figures and images for: Triacylglycerol Storage in Lipid Droplets in Procyclic Trypanosoma brucei
Source: PLoS One. 2014 Dec 10;9(12):e114628. doi: 10.1371/journal.pone.0114628 (PMC4262433; doi:10.1371/journal.pone.0114628)

Figure S1

**A**

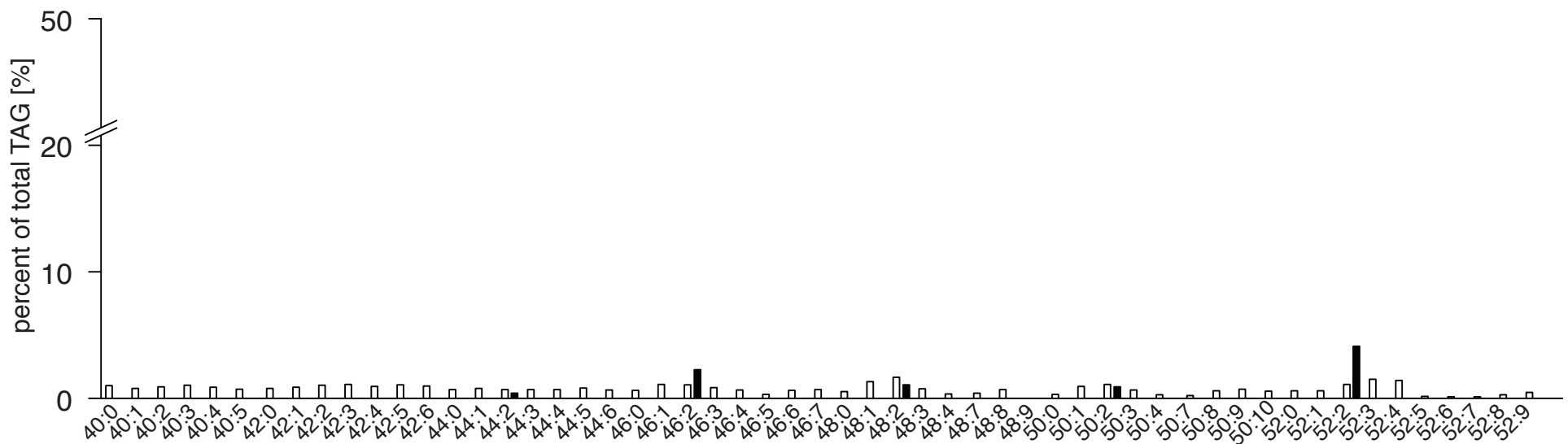

**B**

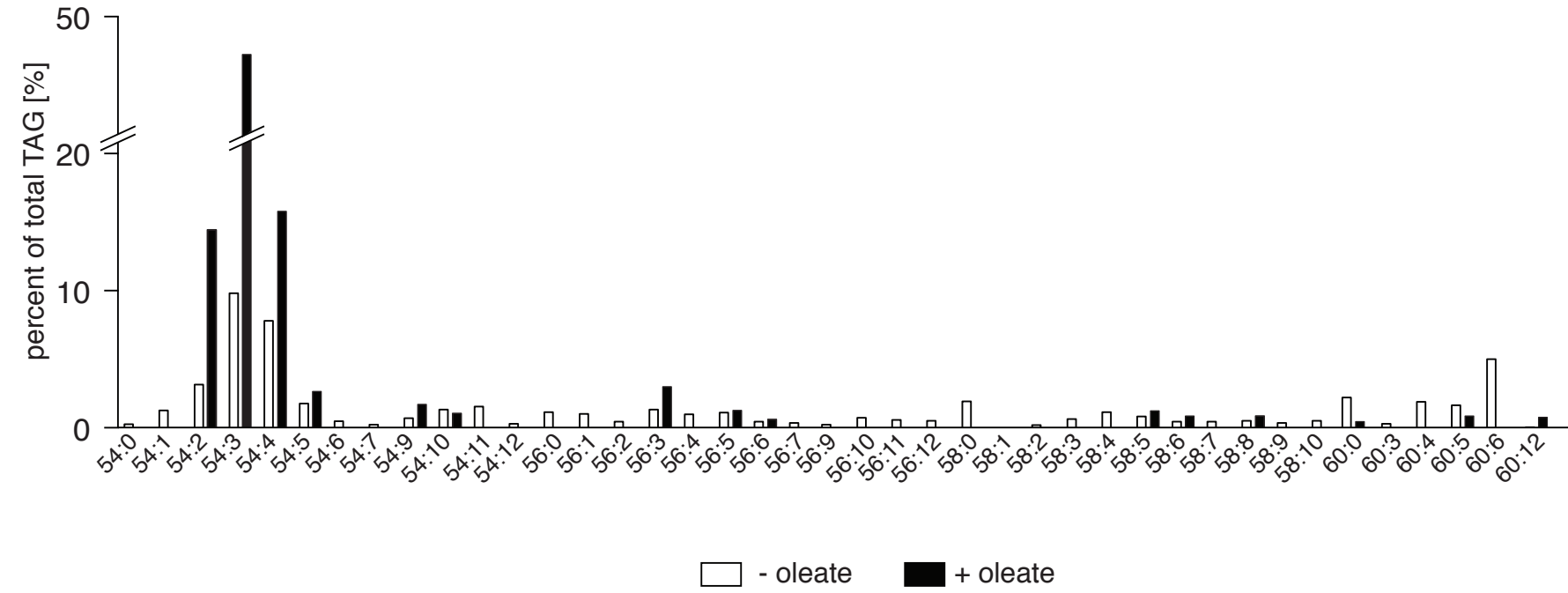

Supplement: S1 Figure — TAG species identified in procyclic T. brucei cells. Relative abundances of TAG species were determined by ESI/MS/MS after oleate feeding for three days (black columns) or in the control (white columns). The nomenclature 54:X indicates the total carbon number of all three acyl chains and the sum of all unsaturated double bonds within the acyl chains. Most TAG species are minor contributions to the total TAG content. (PDF) [file pone.0114628.s001.pdf]

Figure S3

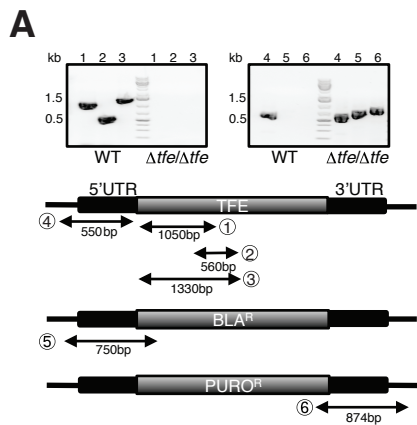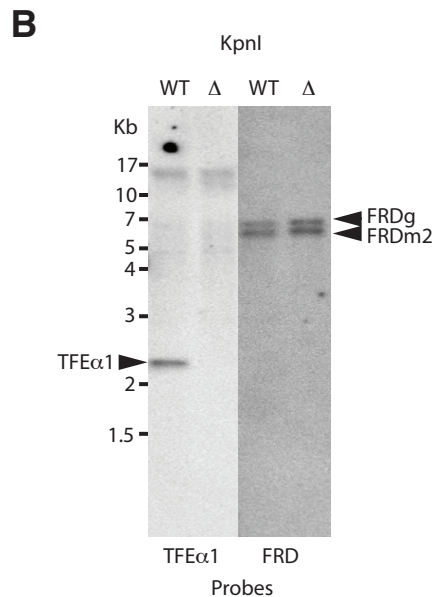

Supplement: S3 Figure — Verification of the Δ tfeα1 /Δ tfeα1 null mutant. (A) Verification of the Δtfeα1/Δtfeα1 null mutant by integration control PCRs. The lanes of the gel are numbered according to the primer combinations used. (B) Southern blot analysis of the Δtfeα1/Δtfeα1 null mutant. Hybridization of KpnI-digested wild-type genomic DNA with the TFEα1 probe revealed the expected 2.2 kb band, whereas loss of this band in the Δtfeα1/Δtfeα1 genomic DNA is diagnostic for loss of the TFEα1 gene. As a control, hybridization of the same blot with the fumarate reductase (FRD) probe showed the identical band pattern in both wild-type and Δtfeα1/Δtfeα1 cell lines, corresponding to the FRDg and FRDm2 genes. DNA fragment sizes are indicated in kilobases (kb). (PDF) [file pone.0114628.s003.pdf]
