# Supplementary material for: Triacylglycerol Storage in Lipid Droplets in Procyclic Trypanosoma brucei
Source: PLoS One. 2014 Dec 10;9(12):e114628. doi: 10.1371/journal.pone.0114628 (PMC4262433; doi:10.1371/journal.pone.0114628)
Supplement: S2 Figure — Alignment of TFEα1 and TFEα2 protein sequences. The TriTrypDB IDs (http://tritrypdb.org/tritrypdb/) of trypanosomatid sequences are LmjF.33.2600 (TFE1-Lm), LmxM.32.2600 (TFE1-Lmex), LbrM.33.2880 (TFE1-Lb), LtaP33.2830 (TFE1-Lt), Tb927.2.4130 (TFE1-Tb), TcIL3000_2_640 (TFE1-Tco), TcCLB.507547.40 (TFE1-TcCLB), LmjF.26.1550 (TFE2-Lm), LmxM.26.1550 (TFE2-Lmex), LbrM.26.1570 (TFE2-Lb), LtaP26.1590 (TFE2-Lt), TcCLB.508981.39 (TFE2-TcCLB). The names of TFEs from other species correspond to their GenBank accession numbers. Gaps (-) were introduced to maximize the alignments. The graphical output of the Clustal alignment was performed with CLC Main Workbench 6. (PDF) [file pone.0114628.s002.pdf]

Figure S2

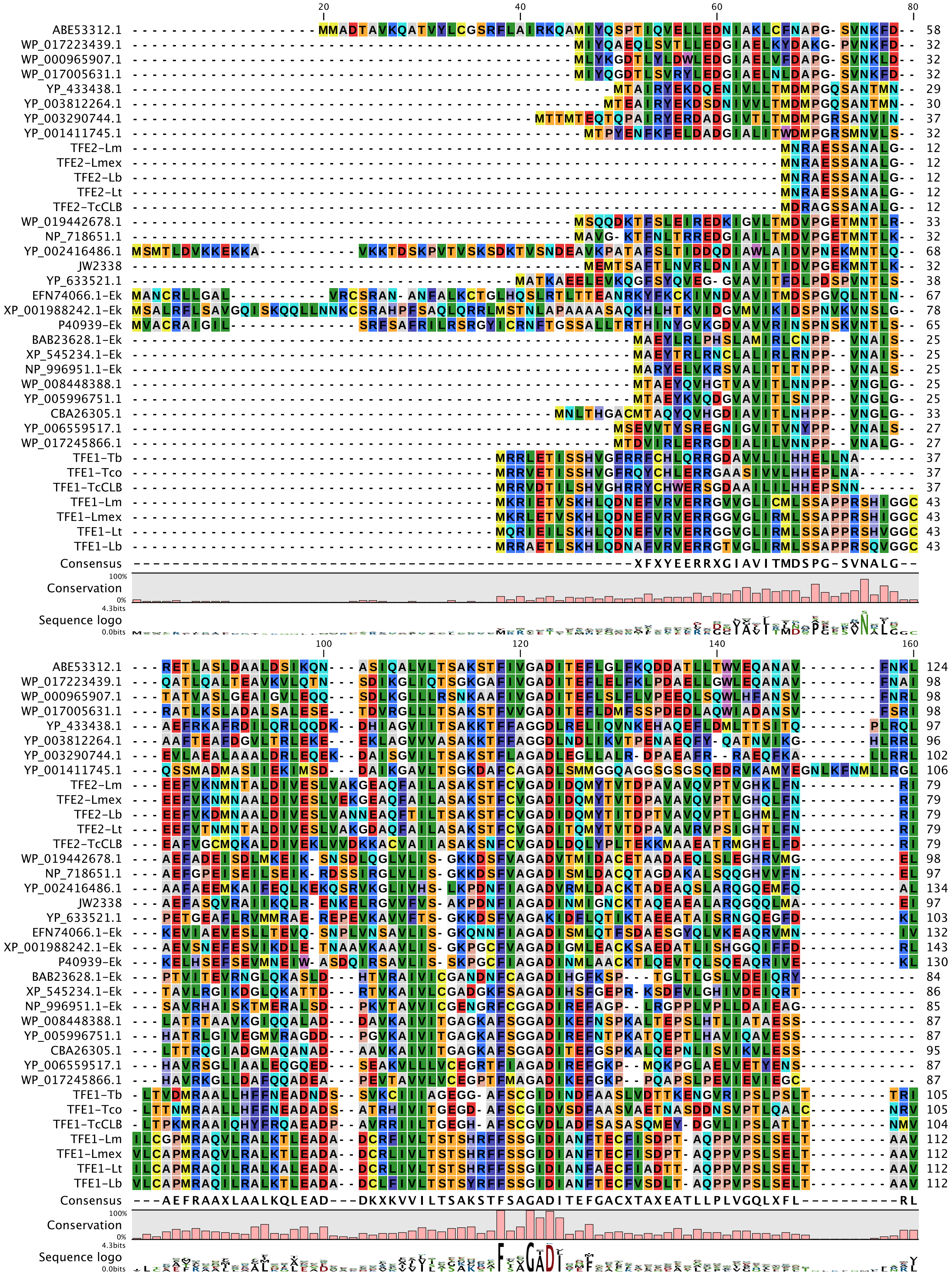

20
40
60
80
ABE53312.1
WP_017223439.1
WP_000965907.1
WP_017005631.1
YP_433438.1
YP_003812264.1
YP_003290744.1
YP_001411745.1
TFE2-Lm
TFE2-Lmex
TFE2-Lb
TFE2-Lt
TFE2-TcCLB
WP_019442678.1
NP_718651.1
YP_002416486.1
JW2338
YP_633521.1
EFN74066.1-Ek
XP_001988242.1-Ek
P40939-Ek
BAB23628.1-Ek
XP_545234.1-Ek
NP_996951.1-Ek
WP_008448388.1
YP_005996751.1
CBA26305.1
YP_006559517.1
WP_017245866.1
TFE1-Tb
TFE1-Tco
TFE1-TcCLB
TFE1-Lm
TFE1-Lmex
TFE1-Lt
TFE1-Lb
Consensus
Conservation
100%
0%
Sequence logo
4.3bits
0.0bits
100
120
140
160
Consensus
Conservation
100%
0%
Sequence logo
4.3bits
0.0bits

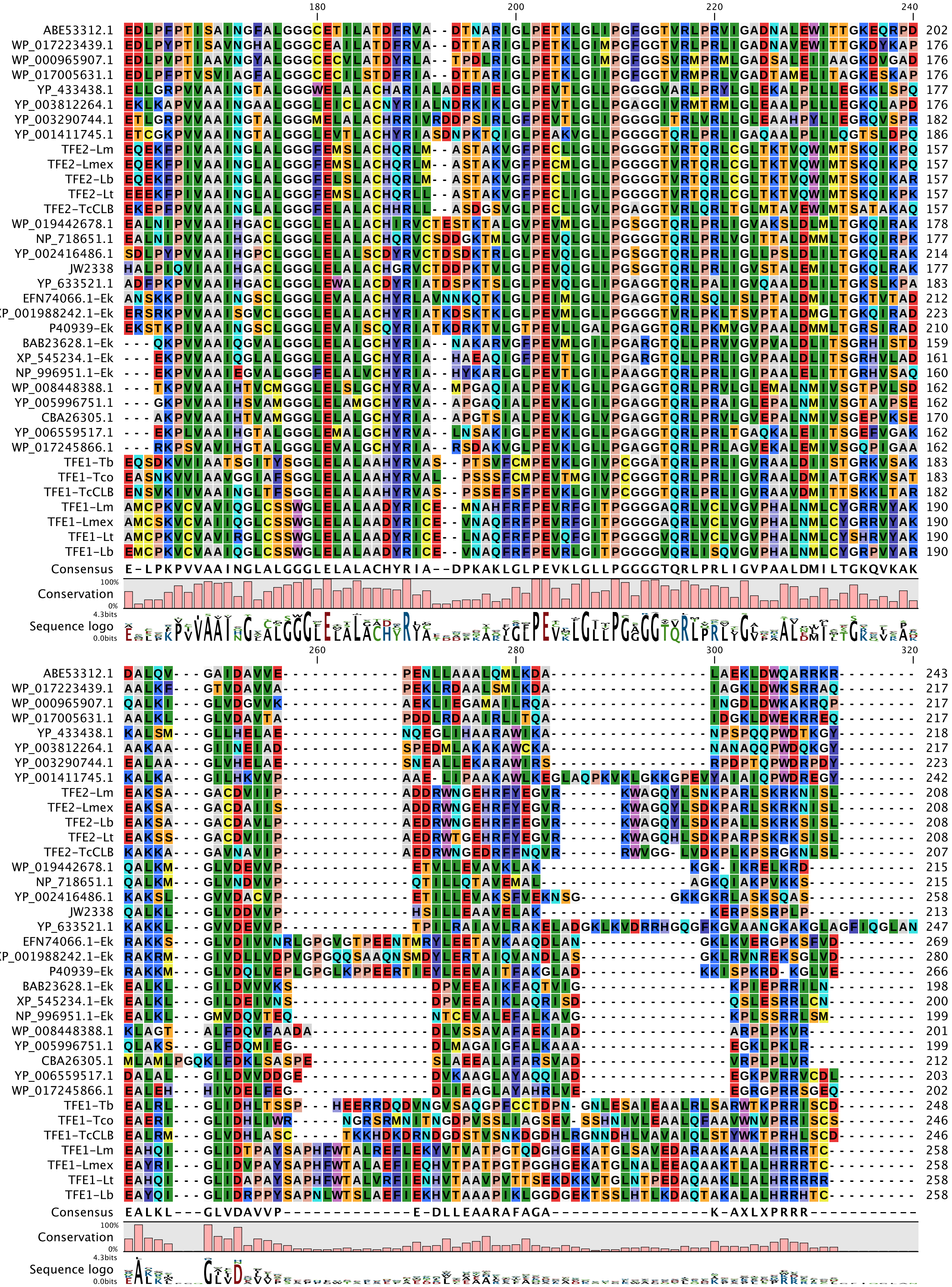
180
200
220
240
ABE53312.1
WP_017223439.1
WP_000965907.1
WP_017005631.1
YP_433438.1
YP_003812264.1
YP_003290744.1
YP_001411745.1
TFE2-Lm
TFE2-Lmex
TFE2-Lb
TFE2-Lt
TFE2-TcCLB
WP_019442678.1
NP_718651.1
YP_002416486.1
JW2338
YP_633521.1
EFN74066.1-Ek
XP_001988242.1-Ek
P40939-Ek
BAB23628.1-Ek
XP_545234.1-Ek
NP_996951.1-Ek
WP_008448388.1
YP_005996751.1
CBA26305.1
YP_006559517.1
WP_017245866.1
TFE1-Tb
TFE1-Tco
TFE1-TcCLB
TFE1-Lm
TFE1-Lmex
TFE1-Lt
TFE1-Lb
Consensus E-LPKPVVAAINGLALGGGLELALACHYRIA--DPKAKLGLPEVKLGLLPGGGGTQRLPRLIGVPAALDMILTGKQVKAK
Conservation
100%
0%
Sequence logo
4.3bits
0.0bits
260
280
300
320
Consensus EALKL---GLVDAVVP------------E-DLLEAARAFAGA---------------K-AXLXPRRR----------
Conservation
100%
0%
Sequence logo
4.3bits
0.0bits

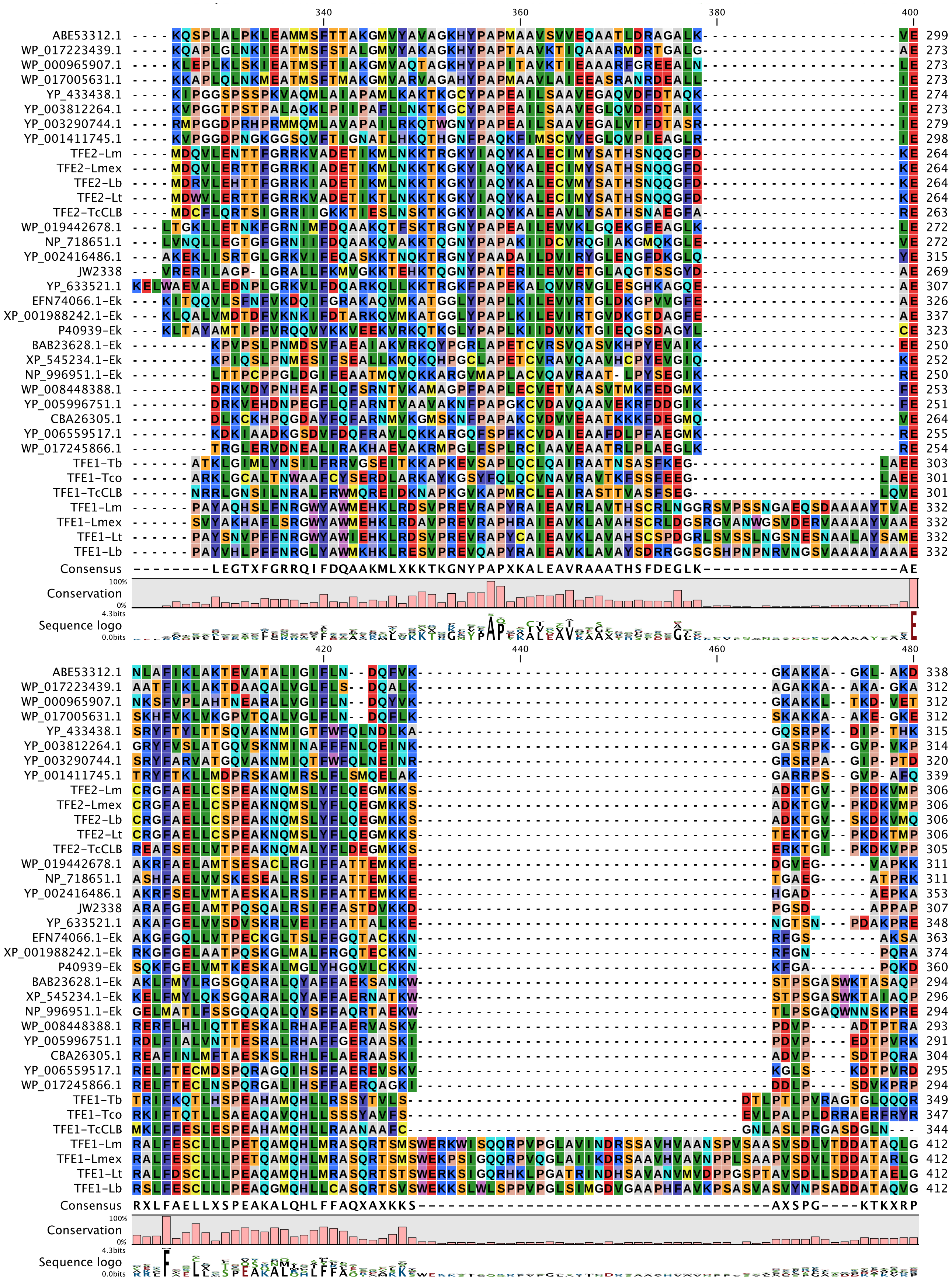

340
360
380
400
ABE53312.1 299
WP_017223439.1 273
WP_000965907.1 273
WP_017005631.1 273
YP_433438.1 274
YP_003812264.1 273
YP_003290744.1 279
YP_001411745.1 298
TFE2-Lm 264
TFE2-Lmex 264
TFE2-Lb 264
TFE2-Lt 264
TFE2-TcCLB 263
WP_019442678.1 272
NP_718651.1 272
YP_002416486.1 315
JW2338 269
YP_633521.1 307
EFN74066.1-Ek 326
XP_001988242.1-Ek 337
P40939-Ek 323
BAB23628.1-Ek 250
XP_545234.1-Ek 252
NP_996951.1-Ek 250
WP_008448388.1 253
YP_005996751.1 251
CBA26305.1 264
YP_006559517.1 255
WP_017245866.1 254
TFE1-Tb 303
TFE1-Tco 301
TFE1-TcCLB 301
TFE1-Lm 332
TFE1-Lmex 332
TFE1-Lt 332
TFE1-Lb 332
Consensus -------LEGTXFGRRQIFDQAAKMLXKKTKGNYPAPXKALEAVRAAATHSFDEGLK------------------AE
Conservation 100% 0%
Sequence logo 4.3bits 0.0bits
420
440
460
480
ABE53312.1 338
WP_017223439.1 312
WP_000965907.1 312
WP_017005631.1 312
YP_433438.1 315
YP_003812264.1 314
YP_003290744.1 320
YP_001411745.1 339
TFE2-Lm 306
TFE2-Lmex 306
TFE2-Lb 306
TFE2-Lt 306
TFE2-TcCLB 305
WP_019442678.1 311
NP_718651.1 311
YP_002416486.1 353
JW2338 307
YP_633521.1 348
EFN74066.1-Ek 363
XP_001988242.1-Ek 374
P40939-Ek 360
BAB23628.1-Ek 294
XP_545234.1-Ek 296
NP_996951.1-Ek 294
WP_008448388.1 293
YP_005996751.1 291
CBA26305.1 304
YP_006559517.1 295
WP_017245866.1 294
TFE1-Tb 349
TFE1-Tco 347
TFE1-TcCLB 344
TFE1-Lm 412
TFE1-Lmex 412
TFE1-Lt 412
TFE1-Lb 412
Consensus RXLFAELLXSPEAKALQHLFFAQXAXKKS---------------------------------AXSPG----KTKXRP
Conservation 100% 0%
Sequence logo 4.3bits 0.0bits

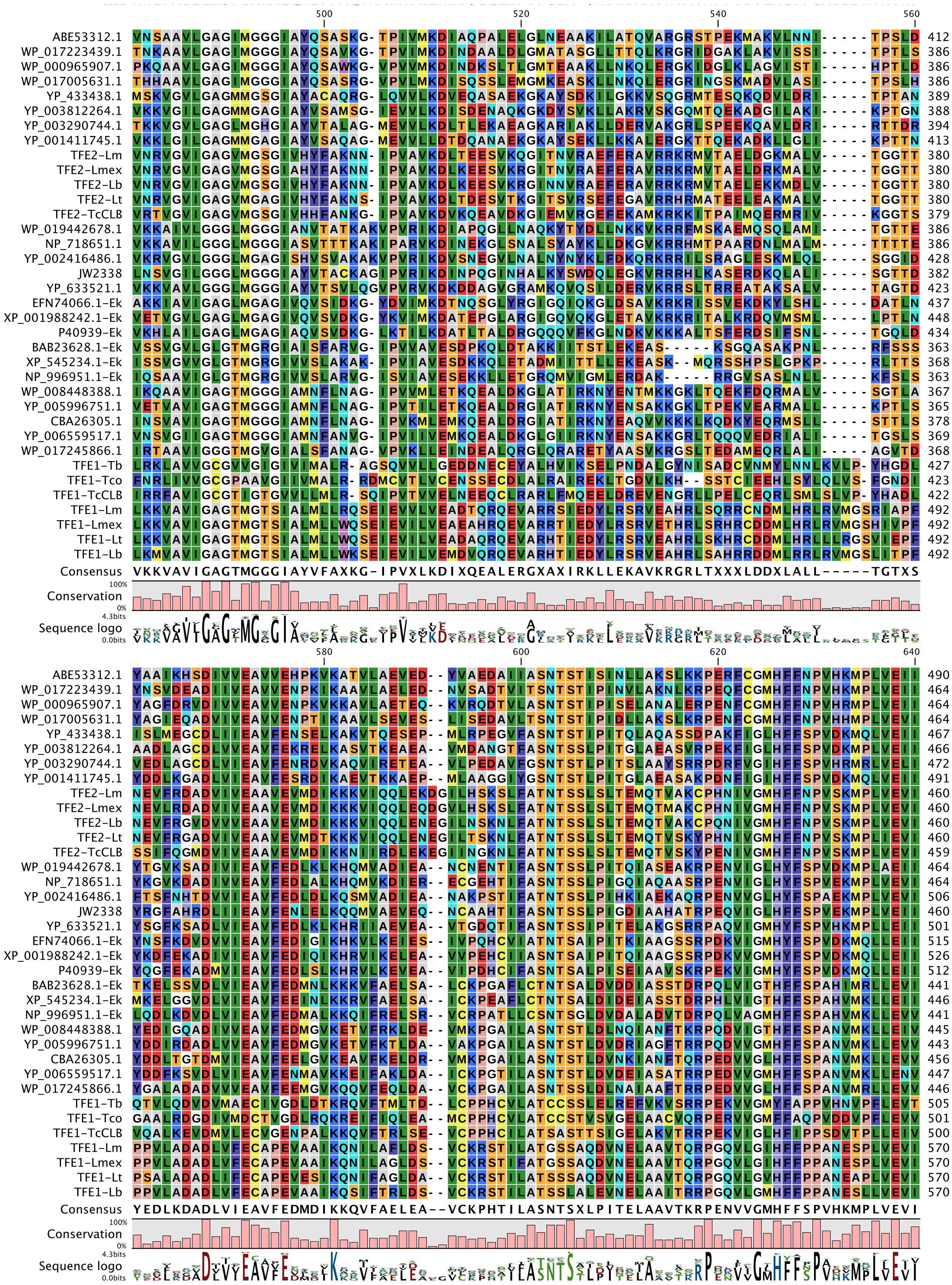
500
520
540
560
ABE53312.1 412
WP_017223439.1 386
WP_000965907.1 386
WP_017005631.1 386
YP_433438.1 389
YP_003812264.1 388
YP_003290744.1 394
YP_001411745.1 413
TFE2-Lm 380
TFE2-Lmex 380
TFE2-Lb 380
TFE2-Lt 380
TFE2-TcCLB 379
WP_019442678.1 386
NP_718651.1 386
YP_002416486.1 428
JW2338 382
YP_633521.1 423
EFN74066.1-Ek 437
XP_001988242.1-Ek 448
P40939-Ek 434
BAB23628.1-Ek 363
XP_545234.1-Ek 368
NP_996951.1-Ek 363
WP_008448388.1 367
YP_005996751.1 365
CBA26305.1 378
YP_006559517.1 369
WP_017245866.1 368
TFE1-Tb 427
TFE1-Tco 423
TFE1-TcCLB 422
TFE1-Lm 492
TFE1-Lmex 492
TFE1-Lt 492
TFE1-Lb 492
Consensus VKKVAVIGAGTMGGGIAYVFAXKG-IPVXLKDIXQEALERGXAXIRKLLEKAVKRGRLTXXXLDDXLALL-----TGTXS
Conservation
100%
0%
Sequence logo
4.3bits
0.0bits
580
600
620
640
ABE53312.1 490
WP_017223439.1 464
WP_000965907.1 464
WP_017005631.1 464
YP_433438.1 467
YP_003812264.1 466
YP_003290744.1 472
YP_001411745.1 491
TFE2-Lm 460
TFE2-Lmex 460
TFE2-Lb 460
TFE2-Lt 460
TFE2-TcCLB 459
WP_019442678.1 464
NP_718651.1 464
YP_002416486.1 506
JW2338 460
YP_633521.1 501
EFN74066.1-Ek 515
XP_001988242.1-Ek 526
P40939-Ek 512
BAB23628.1-Ek 441
XP_545234.1-Ek 446
NP_996951.1-Ek 441
WP_008448388.1 445
YP_005996751.1 443
CBA26305.1 456
YP_006559517.1 447
WP_017245866.1 446
TFE1-Tb 505
TFE1-Tco 501
TFE1-TcCLB 500
TFE1-Lm 570
TFE1-Lmex 570
TFE1-Lt 570
TFE1-Lb 570
Consensus YEDLKDADLVIEAVFEDMDIKKQVFAELEA--VCKPHTILASNTSXLPITELAAVTKRPENVVGMHFFSPVHKMPLVEVI
Conservation
100%
0%
Sequence logo
4.3bits
0.0bits

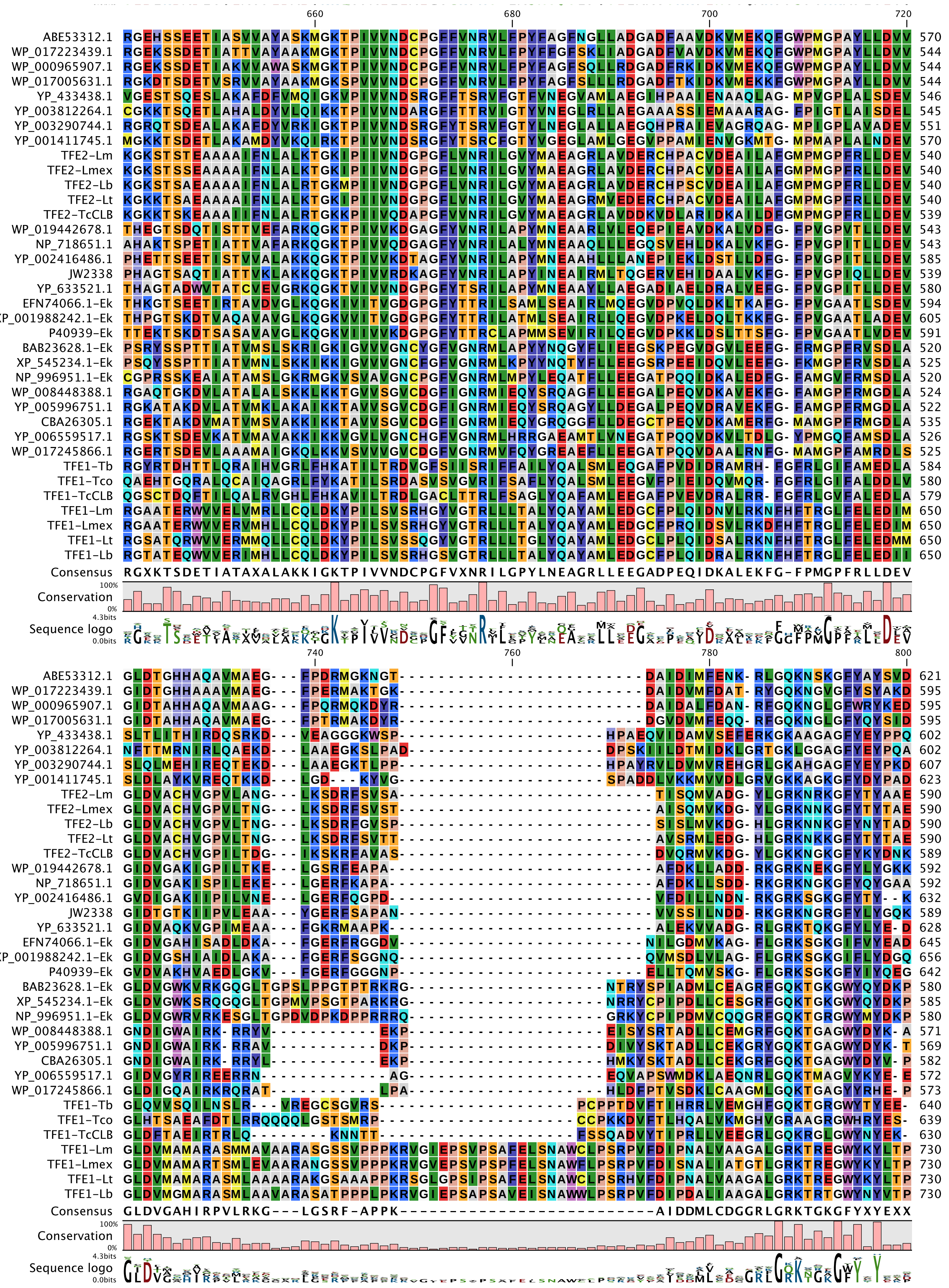
660
680
700
720
ABE53312.1 570
WP_017223439.1 544
WP_000965907.1 544
WP_017005631.1 544
YP_433438.1 546
YP_003812264.1 545
YP_003290744.1 551
YP_001411745.1 570
TFE2-Lm 540
TFE2-Lmex 540
TFE2-Lb 540
TFE2-Lt 540
TFE2-TcCLB 539
WP_019442678.1 543
NP_718651.1 543
YP_002416486.1 585
JW2338 539
YP_633521.1 580
EFN74066.1-Ek 594
XP_001988242.1-Ek 605
P40939-Ek 591
BAB23628.1-Ek 520
XP_545234.1-Ek 525
NP_996951.1-Ek 520
WP_008448388.1 524
YP_005996751.1 522
CBA26305.1 535
YP_006559517.1 526
WP_017245866.1 525
TFE1-Tb 584
TFE1-Tco 580
TFE1-TcCLB 579
TFE1-Lm 650
TFE1-Lmex 650
TFE1-Lt 650
TFE1-Lb 650
Consensus RGXKTSDETIATAXALAKKIGKTPIVVNDCPGFVXNRILGPYLNEAGRLLEEGADPEQIDKALEKFG-FPMGPFRLLDEV
Conservation 100% 0%
Sequence logo 4.3bits 0.0bits
740
760
780
800
ABE53312.1 621
WP_017223439.1 595
WP_000965907.1 595
WP_017005631.1 595
YP_433438.1 602
YP_003812264.1 602
YP_003290744.1 607
YP_001411745.1 623
TFE2-Lm 590
TFE2-Lmex 590
TFE2-Lb 590
TFE2-Lt 590
TFE2-TcCLB 589
WP_019442678.1 592
NP_718651.1 592
YP_002416486.1 632
JW2338 589
YP_633521.1 628
EFN74066.1-Ek 645
XP_001988242.1-Ek 656
P40939-Ek 642
BAB23628.1-Ek 580
XP_545234.1-Ek 585
NP_996951.1-Ek 580
WP_008448388.1 571
YP_005996751.1 569
CBA26305.1 582
YP_006559517.1 572
WP_017245866.1 573
TFE1-Tb 640
TFE1-Tco 639
TFE1-TcCLB 630
TFE1-Lm 730
TFE1-Lmex 730
TFE1-Lt 730
TFE1-Lb 730
Consensus GLDVGAHIRPVLRKG---LGSRF-APPK------------------------AIDDMLCDGGRLGRKTGKGFYXYEXX
Conservation 100% 0%
Sequence logo 4.3bits 0.0bits

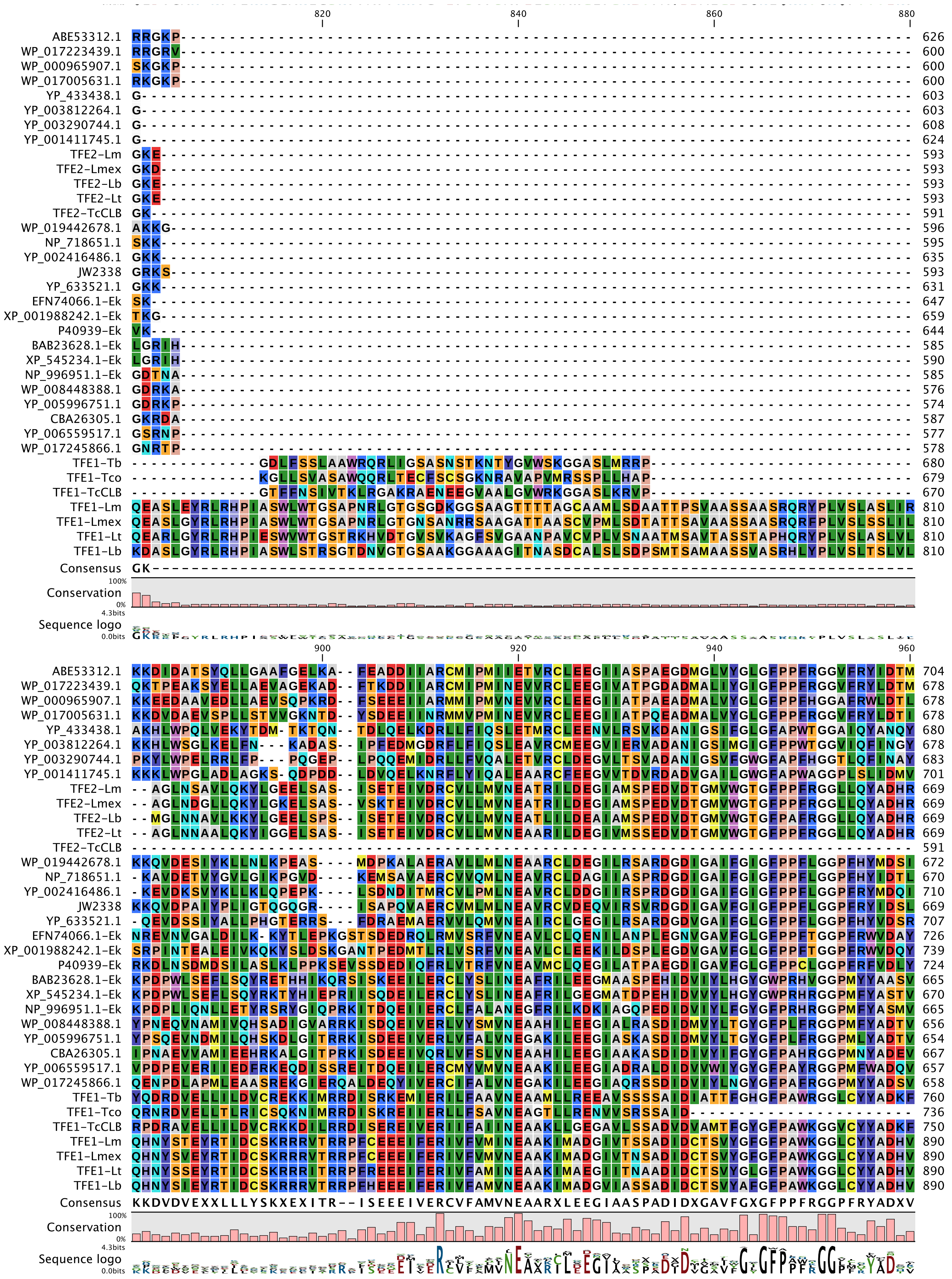
820
840
860
880
ABE53312.1
WP_017223439.1
WP_000965907.1
WP_017005631.1
YP_433438.1
YP_003812264.1
YP_003290744.1
YP_001411745.1
TFE2-Lm
TFE2-Lmex
TFE2-Lb
TFE2-Lt
TFE2-TcCLB
WP_019442678.1
NP_718651.1
YP_002416486.1
JW2338
YP_633521.1
EFN74066.1-Ek
XP_001988242.1-Ek
P40939-Ek
BAB23628.1-Ek
XP_545234.1-Ek
NP_996951.1-Ek
WP_008448388.1
YP_005996751.1
CBA26305.1
YP_006559517.1
WP_017245866.1
TFE1-Tb
TFE1-Tco
TFE1-TcCLB
TFE1-Lm
TFE1-Lmex
TFE1-Lt
TFE1-Lb
Consensus
Conservation
Sequence logo
900
920
940
960
Consensus
Conservation
Sequence logo

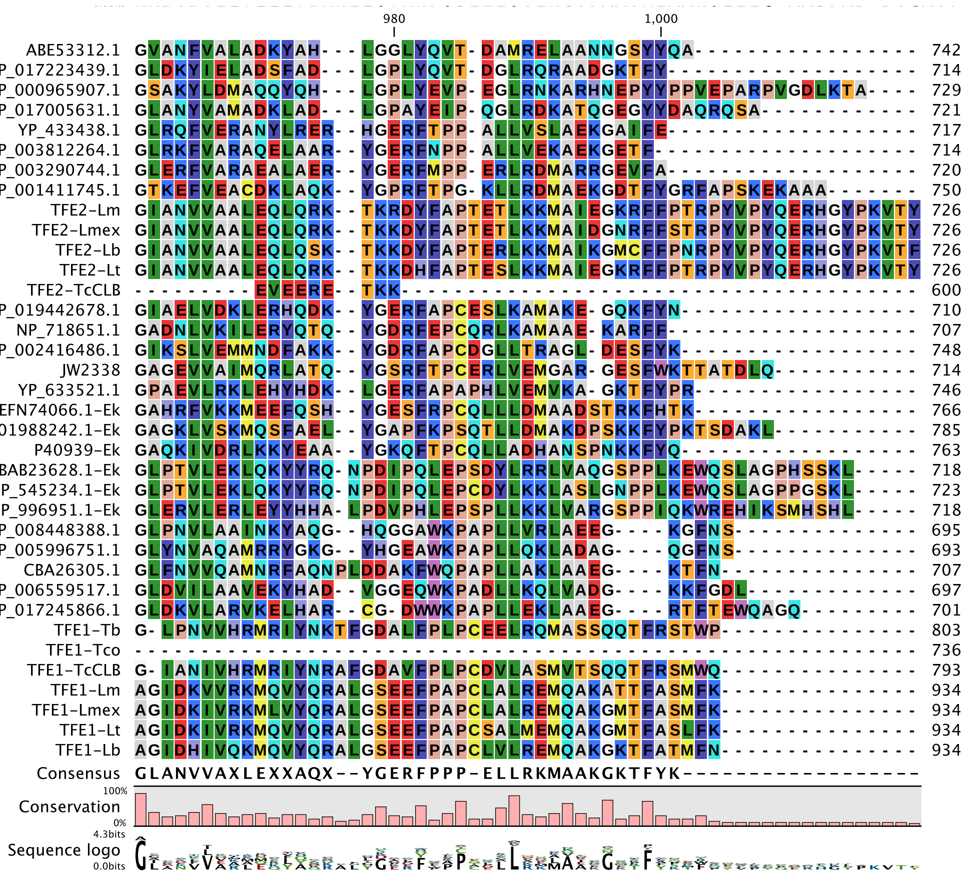
980
1,000
ABE53312.1 GVANFVALADKYAH---LGGLYQVT-DAMRELAANNGSYYQA---------------- 742
WP_017223439.1 GLDKYIELADSFAD---LGPLYQVT-DGLRQRAADGKTFY------------------ 714
WP_000965907.1 GSAKYLDMAQQYQH---LGPLYEVP-EGLRNKARHNEPYYPPVEPARPVGDLKTA---- 729
WP_017005631.1 GLANYVAMADKLAD---LGPAYEIP-QGLRDKATQGEGYYDAQRQSA------------ 721
YP_433438.1 GLRQFVERANYLRER--HGERFTPP-ALLVSLAEKGAIFE------------------ 717
YP_003812264.1 GLRKFVARAQELAAR--YGERFNPP-ALLVEKAEKGETF------------------- 714
YP_003290744.1 GLERFVARAEALAER--YGERFMPP-ERLRDMARRGEVFA------------------ 720
YP_001411745.1 GTKEFVEACDKLAQK--YGPRFTPG-KLLRDMAEKGDTFYGRFAPSKEKAAA------- 750
TFE2-Lm GIANVVAALEQLQRK--TKRDYFAPTETLKKMAIEGKRFFPTRPYVPYQERHGYPKVTY 726
TFE2-Lmex GIANVVAALEQLQRK--TKKDYFAPTETLKKMAIDGNRFFSTRPYVPYQERHGYPKVTY 726
TFE2-Lb GIANVVAALEQLQSK--TKKDYFAPTERLKKMAIKGMCFFPNRPYVPYQERHGYPKVTF 726
TFE2-Lt GIANVVAALEQLQRK--TKKDHFAPTESLKKMAIEGKRFFPTRPYVPYQERHGYPKVTY 726
TFE2-TcCLB ---------EVEERE--TKK--------------------------------------- 600
WP_019442678.1 GIAELVDKLERHQDK--YGERFAPCESLKAMAKE-GQKFYN----------------- 710
NP_718651.1 GADNLVKILERYQTQ--YGDRFEPCQRLKAMAE-KARFF------------------- 707
YP_002416486.1 GIKSLVEMMNDFAKK--YGDRFAPCDGLLTRAGL-DESFYK----------------- 748
JW2338 GAGEVVAIMQRLATQ--YGSRFTPCERLVEMGAR-GESFWKTTATDLQ----------- 714
YP_633521.1 GPAEVLRKLEHYHDK--LGERFAPAPHLVEMVKA-GKTFYPR---------------- 746
EFN74066.1-Ek GAHRFVKKMEEFQSH--YGESFRPCQLLLDMAADSTRKFHTK---------------- 766
XP_001988242.1-Ek GAGKLVSKMQSFAEL--YGAPFKPSQTLLDMAKDPSKKFYPKTSDAKL----------- 785
P40939-Ek GAQKIVDRLKKYEAA--YGKQFTPCQLLADHANSPNKKFYQ----------------- 763
BAB23628.1-Ek GLPTVLEKLQKYYRQ-NPDIPQLEPSDYLRRLVAQGSPPLKEWQSLAGPHSSKL----- 718
XP_545234.1-Ek GLPTVLEKLQKYYRQ-NPDIPQLEPCDYLKKLASLGNPPLKEWQSLAGPPGSKL----- 723
NP_996951.1-Ek GLERVLERLEYYHHA-LPDVPHLEPSPLLKKLVARGSPPIQKWREHIKSMHSHL----- 718
WP_008448388.1 GLPNVLAAINKYAQG--HQGGAWKPAPLLVRLAEEG----KGFNS------------- 695
YP_005996751.1 GLYNVAQAMRRYGKG--YHGEAWKPAPLLQKLADAG----QGFNS------------- 693
CBA26305.1 GLFNVVQAMNRFAQNPLDDAKFWQPAPLLAKLAAEG----KTFN-------------- 707
YP_006559517.1 GLDVILAAVEKYHAD--VGGEQWKPADLLKQLVADG----KKFGDL------------ 697
WP_017245866.1 GLDKVLARVKELHAR--CG-DWWKPAPLLEKLAAEG----RTFTEWQAGQ--------- 701
TFE1-Tb G-LPNVVHRMRIYNKTFGDALFPLPCEELRQMASSQQTFRSTWP--------------- 803
TFE1-Tco -- 736
TFE1-TcCLB G-IANIVHRMRIYNRAFGDAVFPLPCDVLASMVTSQQTFRSMWQ--------------- 793
TFE1-Lm AGIDKVVRKMQVYQRALGSEEFPAPCLALREMQAKATTFASMFK--------------- 934
TFE1-Lmex AGIDKIVRKMLVYQRALGSEEFPAPCLALREMQAKGMTFASMFK--------------- 934
TFE1-Lt AGIDKIVRKMQVYQRALGSEEFPAPCSALMEMQAKGMTFASLFK--------------- 934
TFE1-Lb AGIDHIVQKMQVYQRALGSEEFPAPCLVLREMQAKGKTFATMFN--------------- 934
Consensus GLANVVAXLEXXAQX--YGERFPPP-ELLRKMAAKGKTFYK-----------------
100%
Conservation
0%
4.3bits
Sequence logo
0.0bits
